# A whole-blood cryopreservation method streamlines clinical sample collection for multimodal single-cell immune profiling in sepsis

**DOI:** 10.1172/jci.insight.204610

**Published:** 2026-07-14

**Authors:** Alyssa K. DuBois, Pierre O. Ankomah, Alexis C. Campbell, Renee Hua, Olivia K. Nelson, Christopher A. Zeuthen, M. Kartik Das, Shira Mann, Abigail Mauermann, Blair A. Parry, Nathan I. Shapiro, Michael R. Filbin, Roby P. Bhattacharyya

**Affiliations:** 1 Infectious Disease and Microbiome Program, Broad Institute, Cambridge, Massachusetts, USA.; 2 Division of Infectious Diseases, Department of Medicine, and; 3 Department of Emergency Medicine, Massachusetts General Hospital, Boston, Massachusetts, USA.; 4 Department of Emergency Medicine, Beth Israel Deaconess Medical Center, Boston, Massachusetts, USA.

**Keywords:** Clinical Research, Immunology, Infectious disease, Cellular immune response, Innate immunity, Transcriptomics

## Abstract

Single-cell RNA sequencing (scRNA-seq) of peripheral blood mononuclear cells (PBMCs) has enhanced our understanding of host immune mechanisms in small cohorts, particularly in diseases with complex and heterogeneous immune responses such as sepsis. However, standard PBMC isolation from blood requires technical expertise and over 2 hours of on-site processing using Ficoll density gradient separation (“Ficoll”) for scRNA-seq compatibility, precluding large-scale sample collection at most clinical sites. To minimize on-site processing, we developed cryopreservation with PBMC recovery offsite (Cryo-PRO), a method of immediate on-site whole-blood cryopreservation and subsequent batched PBMC isolation in a central laboratory prior to sequencing. We compared multimodal single-cell immune profiling results from samples processed using Cryo-PRO versus standard on-site Ficoll separation in 23 patients with sepsis. Key outputs, including cell substate fractions, marker genes, and surface protein expression were similar for each method across multiple cell types and substates, including an important monocyte substate enriched in patients with sepsis. Capture of T cell receptor transcripts was also comparable across both methods. Cryo-PRO reduced on-site sample processing time from more than 2 hours to less than 15 minutes and was reproducible across 2 enrollment sites, thus demonstrating potential for expanding multimodal single-cell analyses in multicenter studies of sepsis and other diseases.

## Abstract 2

A new method to easily collect patient blood samples for scRNA-seq without needing lengthy processing by clinical teams to preserve the cells.

## Introduction

Single-cell RNA sequencing (scRNA-seq) has revealed previously unrecognized cell types and transcriptional substates that illuminate pathophysiological variation in many aspects of human disease (1–4). This method has particular appeal for dissecting the cellular basis for heterogeneity in diseases and opening pathways to precision medicine. Sepsis, for example, is a syndrome with expansive differences in clinical course and outcome between patients that is impacted substantially by heterogeneity in patients’ immunological responses. However, transcriptional profiling in patients with sepsis has mostly relied on bulk RNA-seq to generate averaged signatures from all circulating immune cells, obscuring the cellular basis and underlying mechanisms of immune dysfunction (5–9).

In previous work, our group has used scRNA-seq to profile circulating peripheral blood mononuclear cells (PBMCs) in urosepsis, identifying a unique CD14^+^ monocyte subtype (monocyte substate 1, or MS1), that is expanded in sepsis relative to infection without sepsis (10). These monocytes have a gene expression profile similar to myeloid-derived immune suppressor cells, which are immune regulatory cells that inhibit T cell activation, proliferation, and cytotoxic activity (11). MS1 cells may therefore play an immunosuppressive role in sepsis and contribute to important physiological subphenotypes of sepsis, referred to as “endotypes” (12). Several other studies have employed scRNA-seq in small sepsis cohorts (12–15). However, large-scale studies across clinical sites are necessary to appropriately represent the heterogeneity of sepsis with its variable pathogen types, anatomic sites of infection, timing of presentation, severity, trajectory, clinical characteristics, and complex host immune responses. Leveraging the resolution of scRNA-seq at a scale sufficient to capture the major manifestations of sepsis has the potential to enable endotype discovery with sufficient accuracy to impact sepsis care.

Implementing scRNA-seq studies in clinical settings at scale is challenged by several logistical barriers. Blood offers a diverse and dynamic snapshot of the systemic response to infection and is easily collected to investigate immune responses in sepsis and other conditions (16). However, it is necessary to either process samples rapidly before sequencing or employ cryostorage for later analysis to minimize ex vivo transcriptional changes in cells after blood collection. Therefore, processing the blood sample to a point where transcription is halted (e.g., by freezing live cells or fixing them unless sequencing is performed immediately) often falls to operators at the collection site. A common standard approach for scRNA-seq studies of PBMCs involves a density gradient centrifugation step immediately following blood draw (Ficoll-paque processing, or “Ficoll”) to isolate and store immune cells (17). This process, which must be completed promptly at the site of blood collection, is resource-intensive, time-consuming, sensitive to protocol variations, and requires technical skill and training. The complexity of real-time processing of whole-blood samples has limited the widespread use of scRNA-seq in clinical investigations. Moreover, the lack of standardization in processing and analysis can lead to batch effects, hindering comparisons across sites and between studies.

To overcome the practical limitations of scRNA-seq in clinical settings, we developed cryopreservation with PBMC recovery offsite (Cryo-PRO), a method for isolating PBMCs from cryopreserved whole-blood samples. The approach utilizes magnetic depletion of red blood cells followed by fluorescence-activated cell sorting to recover immune cells for scRNA-seq. Cryo-PRO enables the immediate cryopreservation of whole-blood samples at clinical sites, with on-site freezing and storage, allowing for their transfer to a centralized laboratory for PBMC isolation and single-cell profiling. In this study, we directly compare the single-cell transcriptome (scRNA-seq), surface proteome, and single-cell T cell receptor sequencing (scTCR-seq) output from sepsis patient samples processed using Cryo-PRO with those processed by the standard on-site Ficoll gradient separation method. Our findings demonstrate technical equivalence and biological reproducibility between the 2 methods. Cryo-PRO has the potential to enable broad application of multimodal single-cell analysis to multicenter studies and clinical trials by simplifying sample collection and centralizing cell isolation to improve cost efficiency, minimize batch effects, and increase sample sizes.

## Results

Patients greater than 18 years of age who presented to the Emergency Departments of 2 large, academic hospitals in Boston, Massachusetts — Massachusetts General Hospital (MGH) and Beth Israel Deaconess Medical Center (BIDMC) — with clinical concern for sepsis or septic shock with associated organ dysfunction were enrolled in the study. Up to 10 mL of blood was obtained from patients and processing was initiated on site using 2 methods: (a) standard Ficoll gradient separation from whole blood by following standard procedures for isolating and freezing PBMCs (17), followed by storage at –80°C; and (b) Cryo-PRO, by immediately adding dimethyl sulfoxide (DMSO) to a final concentration of 10% (v/v) in 1 mL aliquots of fresh whole blood and freezing at –80°C. To measure potential effects of variation in processing by enrollment site, for a subset of patients, up to 20 mL of blood (separated in two 10-mL tubes) was obtained; 1 tube was immediately couriered to the other clinical site while 1 tube remained at the enrolling site. Processing at both sites using both Ficoll and Cryo-PRO began at the same time upon sample receipt at the receiving site. Blood from 1 healthy donor was obtained from Research Blood Components and processed using both methods as a reference standard. All samples were sent to the Eli and Edythe L. Broad Institute of MIT and Harvard (Broad Institute) for long-term storage at –140°C, subsequent processing, and multimodal single-cell immune profiling.

We adjudicated 23 participants based on the presence of sepsis or bacterial infection for sample processing and sequencing. Septic shock requiring vasopressors for over 24 hours was present in 15 participants (65%), sepsis without shock in 6 participants (26%), and bacterial infection not meeting Sepsis-3 criteria (18) in 2 participants (9%). Bacteremia was present in 7 (30%) of the 23 participants. The median patient age was 66 years (IQR 62.5–76.5), with 35% female. The healthy donor was a 63-year-old male.

**Figure 1:**
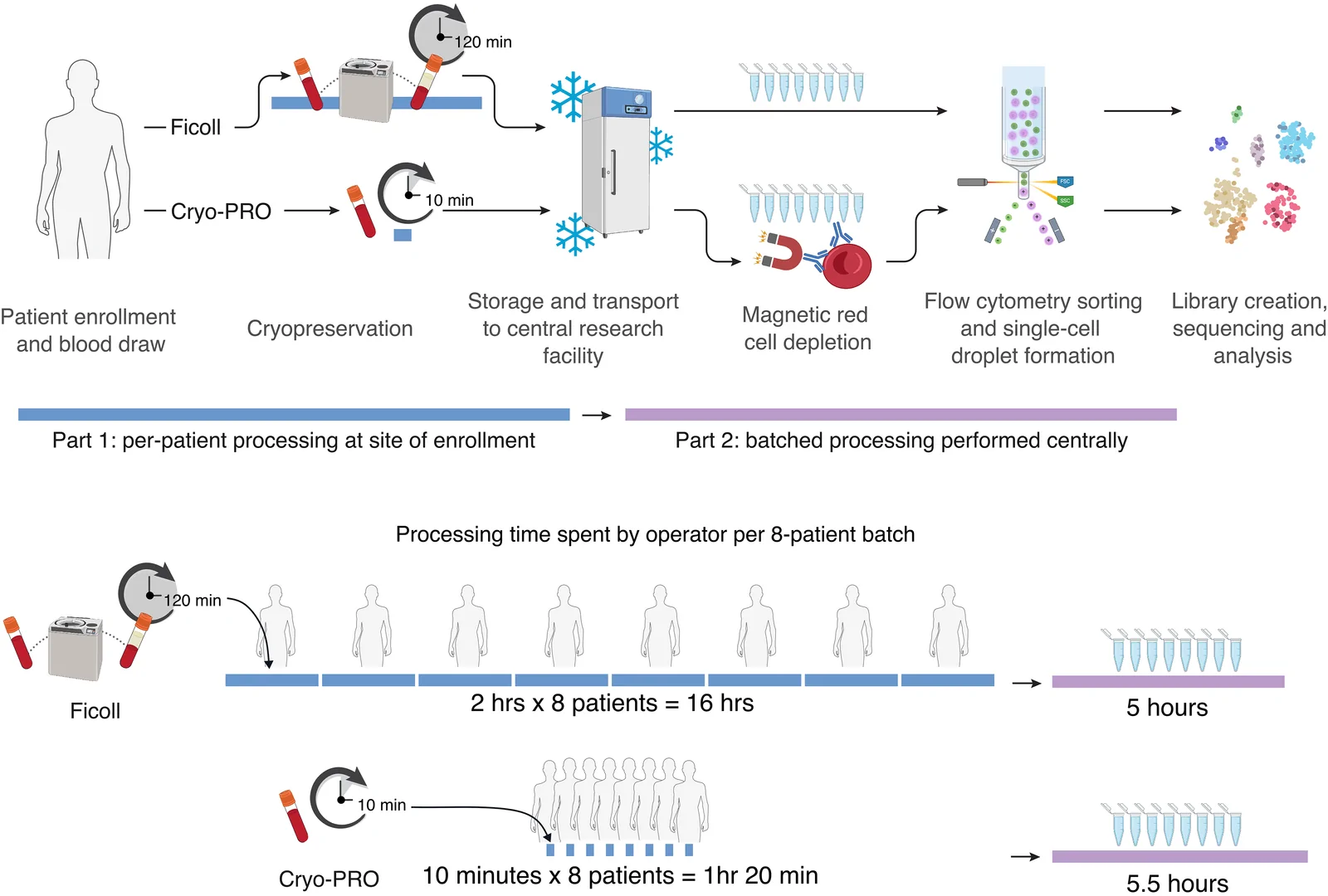
Overview of Ficoll and Cryo-PRO sample processing workflows. Cryo-PRO is designed to expedite sample processing at the site of collection by incorporating a whole-blood cryopreservation step (and subsequent red cell depletion step) to replace standard Ficoll processing. Blue bars denote processing done at the site of enrollment; purple bars denote processing done at a centralized laboratory and include steps up to single-cell droplet formation.

Of the 23 included participants, 8 were processed in parallel at both sites and 15 were processed only at the site of enrollment. Patient-paired frozen Ficoll and Cryo-PRO samples were processed for single-cell analysis at the Broad Institute. Processing included a magnetic red blood cell depletion step (Cryo-PRO samples only), fluorescence-activated cell sorting to recover live PBMCs (DAPI^–^CD45^+^CD235a^–^CD15^–^ cells), and a standard workflow for droplet-based single-cell RNA capture with surface proteome and TCR repertoire measurements (10X Genomics Chromium Next GEM 5′ v2 Kit with cellular indexing of transcriptional epitope sequencing [CITE-seq]) (see Methods) (19). Sample hashing was used to enable pooling of 8 samples per processing batch, and to facilitate postsequencing demultiplexing and multiplet detection. An overview of the sample collection, storage, and processing strategies is summarized in Figure 1.

### Samples processed using Cryo-PRO yield high-quality scRNA-seq and surface proteome data with minimal on-site processing time.

**Figure 2:**
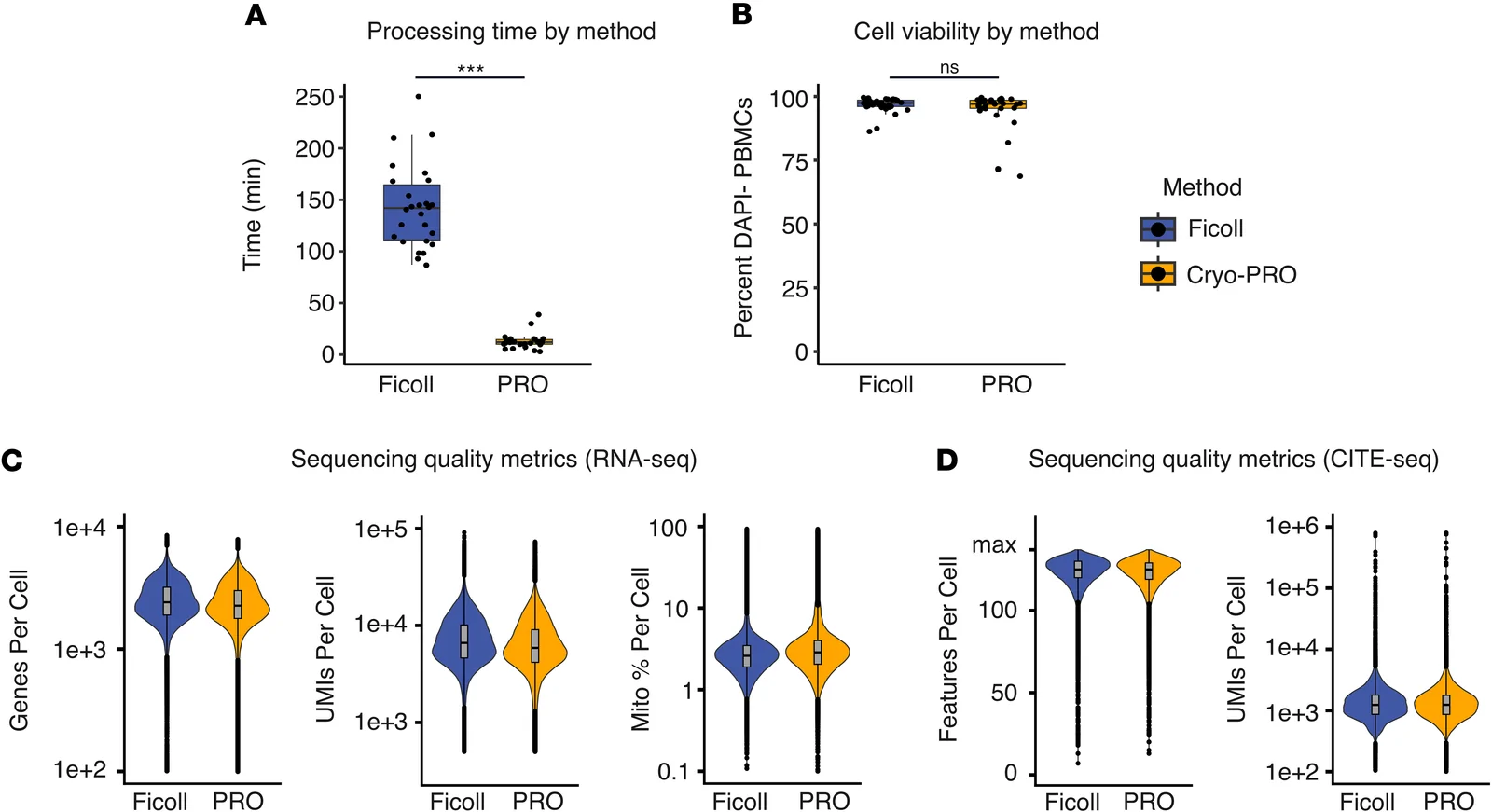
Processing time and quality metrics by cryopreservation method. (**A**) “Hands-on” time spent by operators at clinical sites to process patient samples from initiation of processing after blood draw to placing the sample in a freezer for storage. (**B**) Percentage of CD45^+^CD235a^–^CD15^–^ cells staining DAPI negative on flow cytometry as an indicator of cell membrane integrity and cell viability. Box-and-whisker plots show the median (center line) and interquartile range (IQR; box); whiskers extend to the most extreme values within 1.5 times the IQR. Points represent individual samples. (**C**) Violin plots of RNA-seq quality metrics by method (left to right): unique genes per cell, unique molecular identifiers (UMIs) of RNA transcripts per cell, and percentage of transcripts represented by mitochondrial (Mito) genes per cell. (**D**) Violin plots of CITE-seq quality metrics by method: unique surface protein features (left panel) and UMIs (right panel) per cell (detected via CITE-seq). Violin plots show the distribution of each metric detected per cell; embedded box-and-whisker plots indicate the median and IQR (whiskers = 1.5 × IQR). PRO denotes Cryo-PRO. Statistical significance in **A** and **B** was assessed using a paired, 2-sided *t* test. ***P < 0.001. NS, not significant. According to convention in single-cell data analysis, statistical testing in **C** and **D** was not performed due to the effect of *P*-value inflation (51).

The mean time required for complete on-site processing (from processing start time to storage at –80°C) for Ficoll samples was 2 hours and 23 minutes (SD 40 minutes), while Cryo-PRO samples required an average of 13 minutes (SD 7 minutes) (Figure 2A). The proportion of viable PBMCs recovered by either method was estimated using live/dead staining during flow cytometry sorting (Methods). The mean proportion of live (DAPI^–^) PBMCs (CD235a^–^CD15^–^CD45^+^ cells) was 96.7% (SD 3.0%) for Ficoll samples and 94.1% (SD 8.4%) for Cryo-PRO samples (Figure 2B).

The Cell Ranger pipeline (10X Genomics) was used to process the raw sequencing data, and the Seurat V5 package in R (20) was used for subsequent analysis of single-cell sequencing data (see Methods). Multiplets (cells associated with more than 1 patient hashtag) were removed from analysis. We recovered an average of 2,690 (SD 950) and 2,472 (SD 918) singlet cells per sample for the Ficoll and Cryo-PRO methods, respectively (Supplemental Figure 1; supplemental material available online with this article; https://doi.org/10.1172/jci.insight.204610DS1). Because Ficoll input volumes varied and were not recorded precisely, these values are descriptive and should not be interpreted as a direct comparison of recovery efficiency between methods. Sequencing quality was assessed using the following standard metrics: (a) number of genes sequenced per cell, (b) number of unique molecular identifiers (UMIs) per cell, and (c) percentage of mitochondrial genes sequenced per cell. Higher numbers of genes per cell and UMIs per cell indicate greater per-cell transcript recovery, while a greater percentage of mitochondrial genes suggests cell damage (21). Quality metrics showed similar distributions between methods (Figure 2C). The majority of cells (97.6% from Ficoll processing and 94.4% from Cryo-PRO) passed commonly used quality thresholds (>250 genes per cell, >1,000 UMIs, and <10% mitochondrial genes) (22). Detection of antibody-derived tags (ADTs) used for CITE-seq surface proteome measurement was similar between the 2 methods (Figure 2D). Quality metrics were similar between methods at an individual-patient level (Supplemental Figure 2).

### Cryo-PRO enables identification of transcriptional substates, gene expression patterns, and surface protein markers.

**Figure 3:**
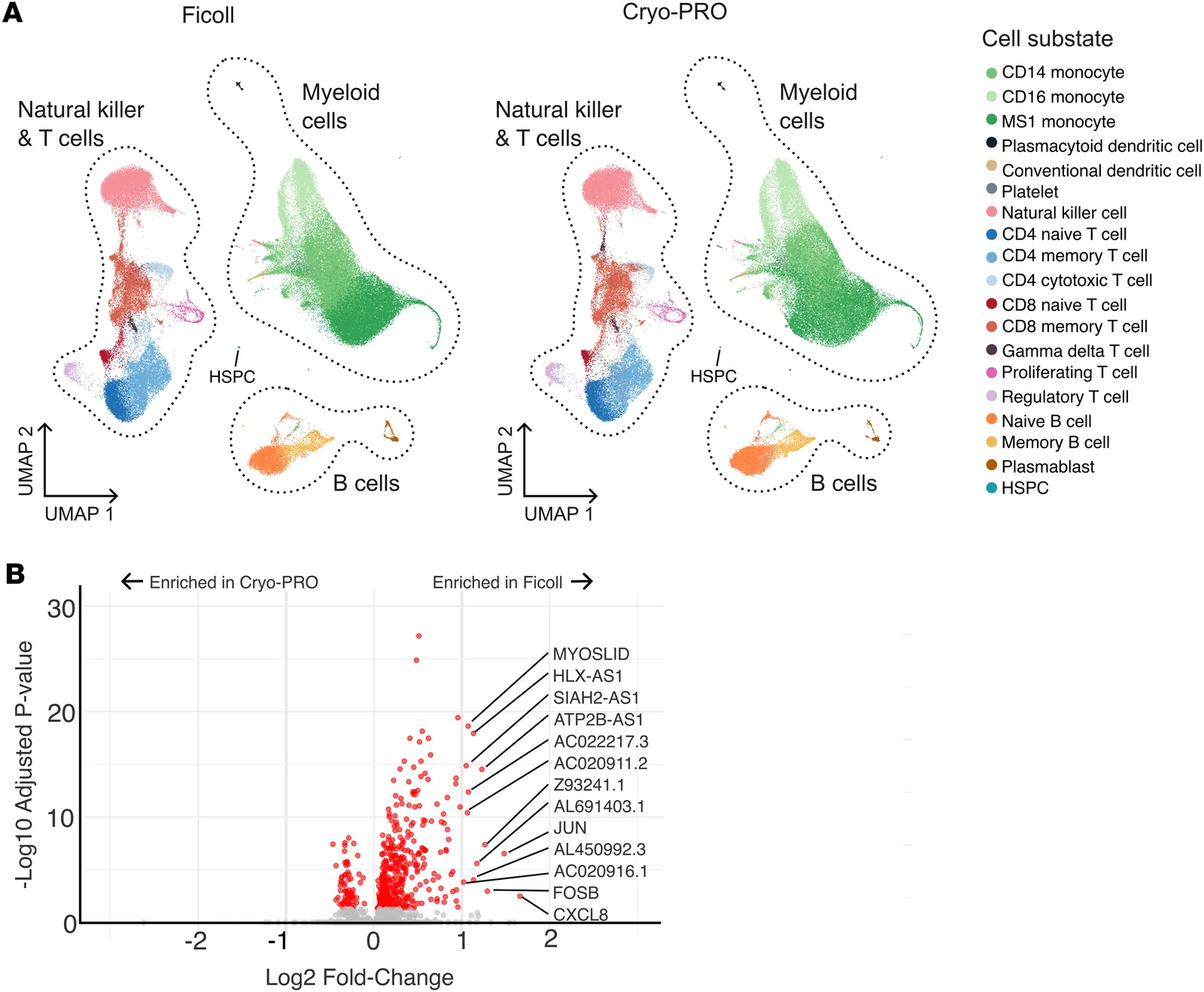
Comparison of cell substate identification and gene expression by method. (**A**) Two-dimensional uniform manifold approximation and projection (UMAP) of cells by processing method: Ficoll (left) and Cryo-PRO (right). Dotted outlines represent types of PBMCs. Cell substates were identified by clustering cells of each method independently; substate identities were then projected onto a shared set of UMAP axes (see Methods). HSPC, hematopoietic stem and progenitor cell. (**B**) Volcano plot showing genes differentially upregulated (positive log_2_[fold change]) or downregulated (negative log_2_[fold change]) in Ficoll compared with Cryo-PRO using pseudobulk gene expression data from all cells (see Methods). Genes with adjusted *P* values of less than 0.05 are shown in red; those with *P* < 0.05 and abs(log_2_[fold change]) > 1 are labeled.

We next assessed whether Cryo-PRO supports scRNA-seq and surface proteome datasets of sufficient quality to reproduce biologically relevant results compared to Ficoll. scRNA-seq analysis was performed separately for cells obtained from each processing method (86,083 cells for Ficoll and 79,089 cells for Cryo-PRO) to ensure independent identification of cell identity and gene expression patterns (see Methods). Clusters enriched for dead and dying cells, identified by the predominance of mitochondrial genes and poor sequencing depth, were removed from further analysis as an additional quality control measure (23). These amounted to 3,602 cells from Ficoll (4.2% of total) and 5,712 cells from Cryo-PRO (7.2% of total) (*P* = 0.003, paired 2-sided *t* test). We identified transcriptionally similar cells that expressed canonical marker genes for the major mononuclear immune cell types (i.e., T cells, B cells, natural killer cells, monocytes, and dendritic cells). Subclustering within each cell type identified higher-resolution clusters of cells with additional transcriptional similarity (i.e., cell substates, e.g., CD4^+^ memory T cells, naive B cells, etc.), which were classified by comparison with reference datasets (20). All the major mononuclear immune cell types, comprising a total of 17 cell substates, were identified from cells isolated using either Ficoll or Cryo-PRO, each clustered independently but projected onto a shared set of 2-dimensional uniform manifold approximation and projection (UMAP) axes for visualization (Figure 3A; see Methods).

**Figure 4:**
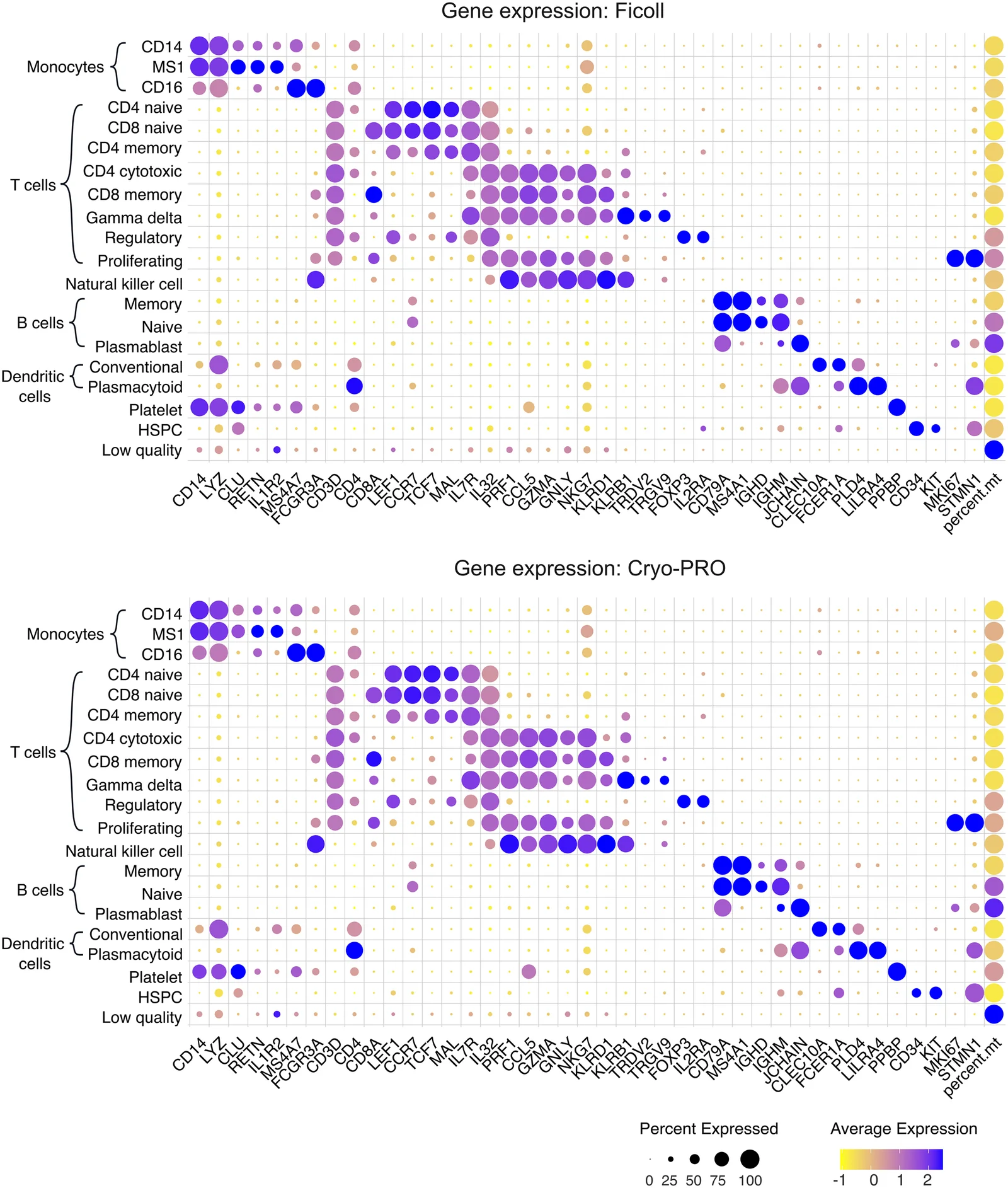
Dot plots of key marker genes and percent mitochondrial reads (percent.mt) for cell substates identified in scRNA-seq analysis by method (top, Ficoll; bottom, Cryo-PRO). Color represents scaled relative expression (blue = higher expression), and size represents the proportion of cells in each substate where the feature was detected.

**Figure 5:**
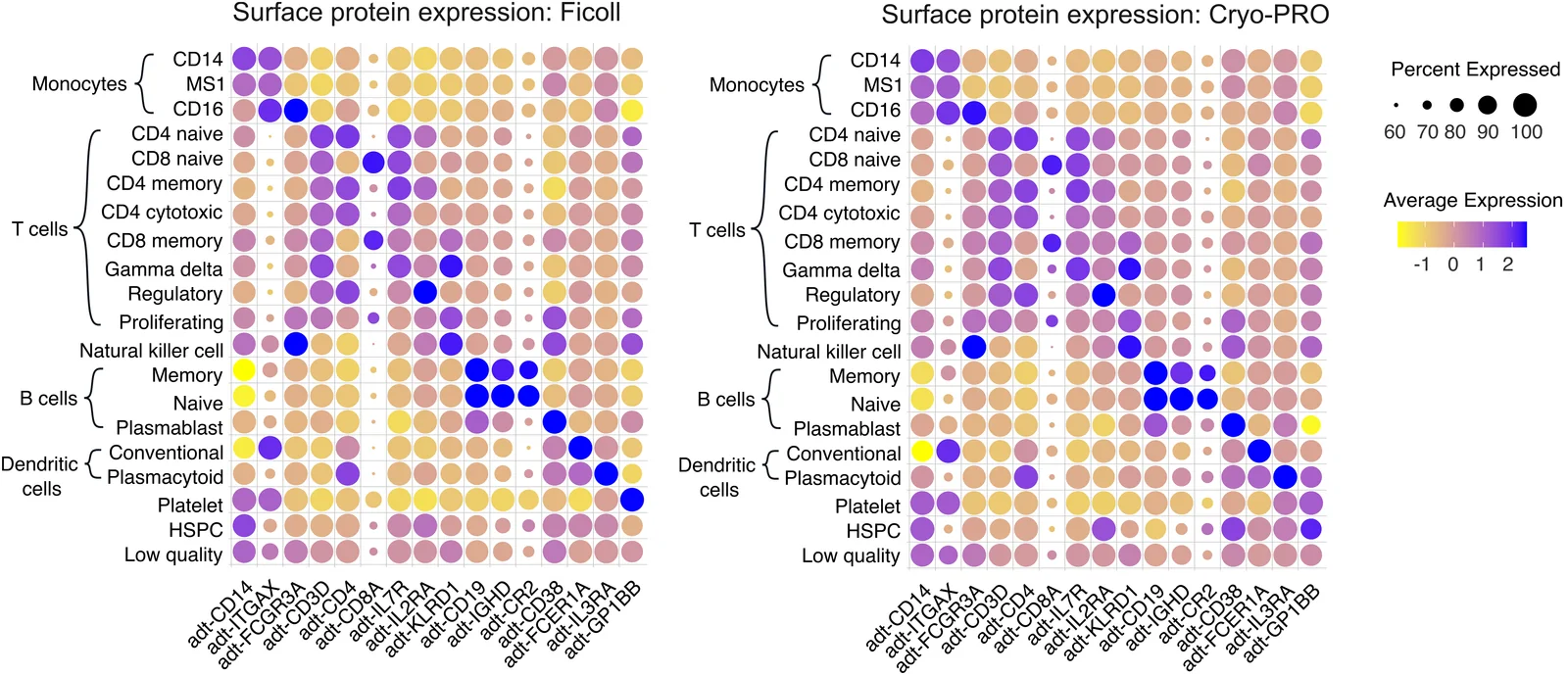
Dot plots of key surface marker proteins (detected using CITE-seq) for each cell substate. Color represents scaled relative expression (blue = higher expression), and size represents the proportion of cells in each substate where the feature was detected.

Top marker genes distinguishing each cluster were identified using the FindMarkers function in Seurat (20); rank was determined by fold change in gene expression within cells of each cluster compared to the cells outside of the cluster (24) (Supplemental Table 1). The average expression of key identifying genes (Figure 4, color scale) and cell surface proteins (Figure 5, color scale) was similar between Ficoll and Cryo-PRO methods for each cell type and substate, as was the proportion of cells for which these features could be detected (Figures 4 and 5, dot size). Our analysis identified the MS1 cell substrate, which was previously discovered to be enriched in patients with sepsis (10, 25). Expression patterns for key MS1 marker genes were similar between Ficoll and Cryo-PRO samples (Supplemental Figure 3A).

In an orthogonal approach, we used FindMarkers and the DESeq2 package in R to compare gene expression between all cells processed by the 2 methods to identify differentially expressed genes (Supplemental Table 2). Among statistically significant (*P*_adj_ < 0.05) genes, we did not observe substantial (greater than 4) fold-change differences in expression between the 2 methods. Most genes with more than a 2-fold expression change were non-coding genes; exceptions included CXCL8, FOSB, and JUN, which were slightly upregulated in Ficoll samples (Figure 3B). Similar differentially expressed genes were identified when comparisons were performed within each major cell type (Supplemental Figure 3B), rather than across all cells. Crucially, no genes used to identify cell types were differentially expressed by more than a 2-fold change. Pathway analysis could not be performed due to the sparse number of robustly differentially expressed genes. However, immediate early genes are a class of genes commonly transiently upregulated in many types of cells as a primary response to a variety of stimuli; the presence of the immediate early genes JUN and FOSB may suggest an early response to ex vivo stimulation in Ficoll-processed cells (26, 27).

### Ficoll and Cryo-PRO yield similar immune cell type and substate abundances.

Defining the composition of circulating immune cells and their substates on a per-patient level is an informative application of scRNA-seq. In sepsis, heterogeneity in the distribution of immune cell types and states between patients is thought to contribute to differences in illness trajectory, outcomes, and response to therapies (28). To evaluate agreement between methods for characterizing immune cell profiles in sepsis, we computed proportions for each cell type and substate for each sample processed using both Ficoll and Cryo-PRO methods. We defined proportion of cell type as the number of cells of a particular type (e.g., B cells) divided by the number of all PBMCs combined. For cell substates, proportion was defined as the number of cells assigned to a substate divided by the total number of cells of that cell type (e.g., number of CD16^+^ monocytes divided by the total number of monocytes). We then compared cell type proportions between paired Ficoll and Cryo-PRO samples from each of the participants using correlations. Substate proportions were calculated for monocytes, T cells, B cells, and dendritic cells; natural killer cells did not demonstrate unique substates, and substate proportions were therefore not computed.

**Figure 6:**
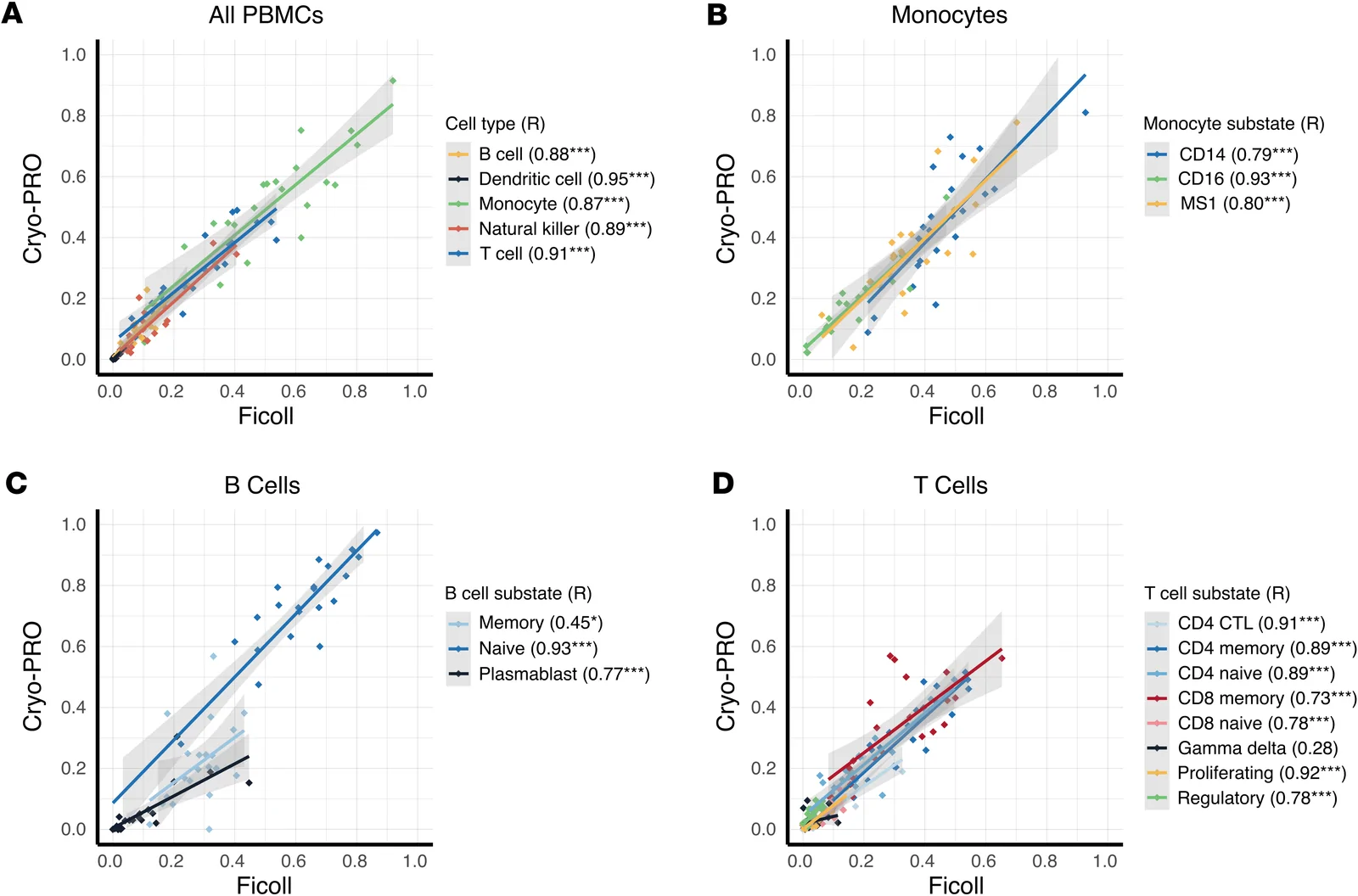
Correlation of cell type and substate proportions between Ficoll and Cryo-PRO methods. (**A**) Scatter plot of cell type proportion from Ficoll and Cryo-PRO. Each point represents the proportion of one cell type from one patient sample, as measured by each method. Each cell type is represented by a different color. Proportion is the number of cells of one cell type divided by the total number of PBMCs from that patient sample. Patient-paired Ficoll:Cryo-PRO samples are plotted to assess correlation by method for each patient. (**B**–**D**) Scatter plots of cell substate proportion from Ficoll and Cryo-PRO. Each point represents the proportion of one cell substate from one patient sample, as measured by each method. Each cell substate is represented by a different color. Proportion is the number of cells of one cell substate divided by the total number of cells from its cell type from that patient sample. Patient-paired Ficoll:Cryo-PRO samples are plotted to assess correlation in method for each patient. Trendlines indicate a linear regression fit with shaded 95% confidence intervals. Pearson’s correlations (*R*) are shown for all trendlines. *P < 0.05, ***P < 0.001.

Proportions of cell types were significantly correlated between methods (Figure 6A), with Pearson correlations (*R*) ranging between 0.87 and 0.95 (*P* < 0.001 for all comparisons). For most substates, correlations were also positive and statistically significant (*R* values from 0.45 to 0.95; *P* < 0.05), except for γδ T cells (*R* = 0.28, not significant [NS]) (Figure 6, B–D, and Supplemental Figure 4). γδ T cells were present at very low counts across methods, likely increasing the effects of outliers on correlations. Within monocytes, correlations across methods were higher for CD16^+^ monocytes (*R* = 0.93, *P* < 0.001) than for CD14^+^ MS1 (*R* = 0.80, *P* < 0.001) and classical CD14^+^ (*R* = 0.79, *P* < 0.001). Comparison of cell substate proportions between Ficoll and Cryo-PRO at an individual-participant level showed a high degree of similarity across each of the 24 participants analyzed (Supplemental Figure 5 and Supplemental Table 3), with especially close alignment for the 15 patients whose samples were processed immediately at clinical sites (Supplemental Figure 5A).

**Figure 7:**
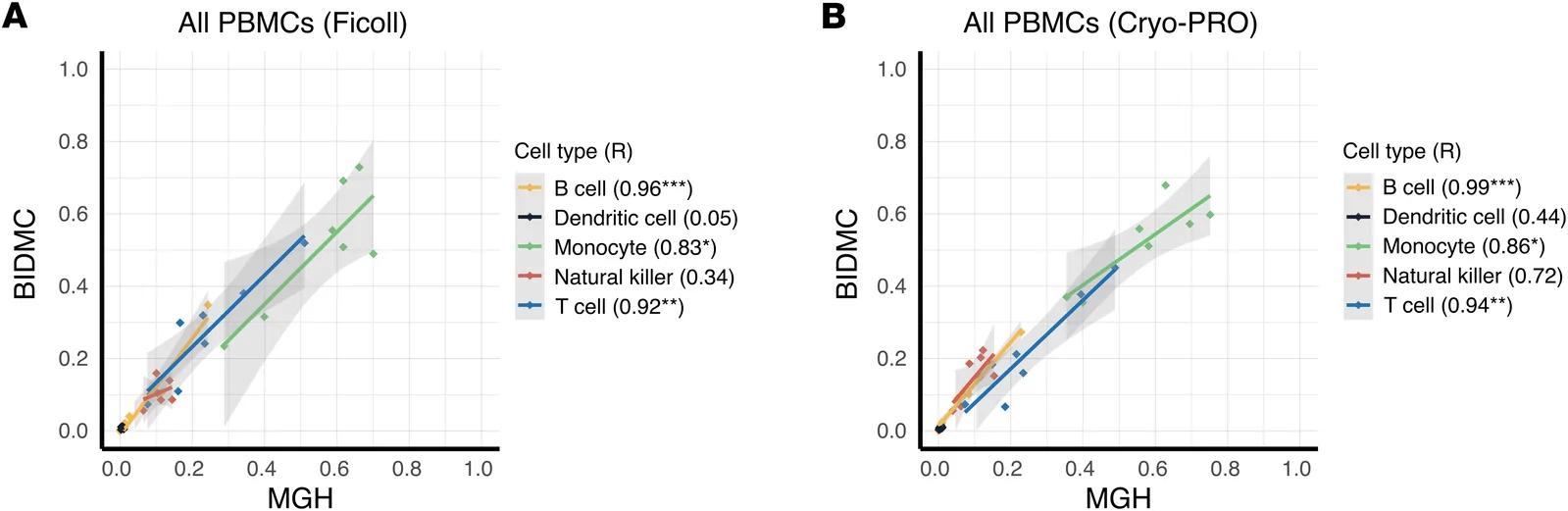
Between-site reproducibility of cell type proportion for samples processed at both locations. Patient-paired Ficoll:Ficoll samples (**A**) and Cryo-PRO:Cryo-PRO samples (**B**) from the 2 different enrollment sites are plotted to assess between-site technical reproducibility. Each point represents the proportion of one cell type from one patient sample, processed at each site. Each cell type is represented by a different color. Proportion is the number of cells of 1 cell type divided by the total number of PBMCs from that patient sample. Trendlines indicate a linear regression fit with shaded 95% confidence intervals. Pearson’s correlations (R) are shown for all trendlines. *P < 0.05, ** *P* < 0.01, ***P < 0.001.

To evaluate the robustness of Cryo-PRO to processing location, we assessed the technical reproducibility of scRNA-seq results for the 8 patients whose samples were processed simultaneously at MGH and BIDMC, acknowledging inherent processing delays due to sample transport between sites (see Methods). Proportions of major cell types (monocytes, B cells, and T cells) were highly correlated between sites for Ficoll (*R* values from 0.83 to 0.96, *P* < 0.001; Figure 7A) and for Cryo-PRO (*R* values from 0.86 to 0.99, *P* < 0.001; Figure 7B). Dendritic cells (Ficoll *R* = 0.05, Cryo-PRO *R* = 0.44; both NS) and natural killer cells (Ficoll *R* = 0.34, Cryo-PRO *R* = 0.72; both NS) were poorly correlated between sites, possibly due to small overall cell counts and variable yield between processing runs that exaggerate differences in cell proportions. Correlations were significant for nearly all substates of monocytes, T cells, and B cells, and dendritic cells for each method between sites (Supplemental Figure 6), although for some substates including MS1, cross-site correlations were slightly lower for Cryo-PRO than Ficoll.

### scTCR-seq yields comparable repertoire capture from Ficoll and Cryo-PRO samples.

T cell lymphopenia and altered TCR diversity are recognized features of sepsis and its recovery. Previous studies have demonstrated reduced TCR repertoire breadth early in the course of septic shock (29, 30), with persistent contraction of the repertoire associated with increased mortality, higher rates of nosocomial infection (29), and reactivation of latent viral infections such as cytomegalovirus (30). These observations underscore the clinical importance of tracking TCR repertoire dynamics in sepsis. Capturing these data alongside paired single-cell gene expression data provides valuable insight into immune dysfunction within cellular substates, as well as gene expression programs associated with changes in clonotype diversity.

To determine whether paired single-cell transcriptomic and TCR profiling are preserved with Cryo-PRO, we applied the 10X Genomics 5′ v2 Immune Profiling workflow (Methods) to matched patient samples processed using either Ficoll or Cryo-PRO. This strategy allows for joint recovery of full-length V(D)J sequences and gene expression data from the same cells. We compared TCR recovery and clonotype representation across both methods.

**Figure 8:**
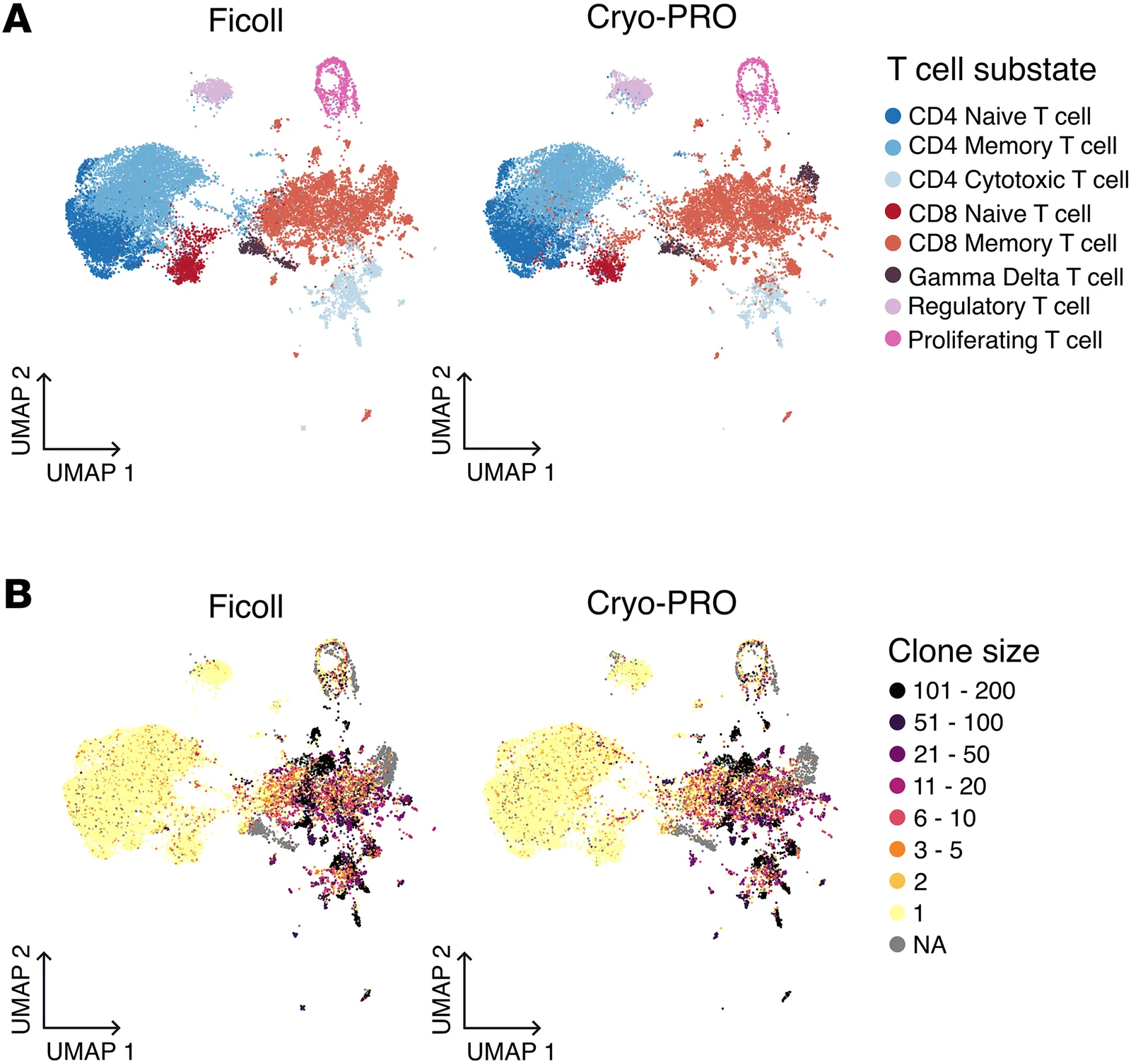
T cell substates and corresponding TCR repertoire clonality by method. UMAP of T cells profiled, split by method (left, Ficoll; right, Cryo-PRO). Cells are colored by (**A**) their cell substate, or by (**B**) the number of identical TCR sequences to represent clone size (darker = greater clonal expansion).

Among Ficoll-processed samples, we recovered TCR sequences from 21,876 cells, representing 98.6% of T cells with transcriptomic data. For Cryo-PRO samples, we recovered TCR sequences from 18,447 cells (96.5% of T cells with transcriptomic data). Expanded and unique clonotypes were represented similarly on UMAP projections of Ficoll and Cryo-PRO T cells (Figure 8). As expected, effector and cytotoxic cells (CD4^+^ cytotoxic T cells and CD8^+^ memory T cells), which expand during acute immune responses to infection (31, 32), displayed the highest levels of clonal expansion by both methods, whereas naive CD4^+^ and CD8^+^ T cell substates displayed greater sequence diversity and fewer expanded clonotypes (Figure 8).

Per-patient clonotypes were generally similar between methods in the proportion of expanded clones (Supplemental Figure 7). Discrepancies in clonal proportions were generally attributable to low T cell recovery from at least one of the samples (Supplemental Figure 7). For patients with samples processed at both clinical sites, we observed similar trends, with clonal proportions closely resembling each other between processing center and method for each patient profiled (Supplemental Figure 7).

We next compared exact nucleotide sequences of the captured TCR clonotype transcripts between methods. Our expectation was that while many clonotypes could be present at very low frequencies in a given sample, expanded clonotypes would be detected at higher frequencies in both Ficoll and Cryo-PRO samples from the same patient. For each patient, we calculated the abundance of each matching unique TCR sequence as a proportion of the total TCR sequences and compared these proportions between methods and processing centers. Patient-matched Ficoll and Cryo-PRO TCR sequence proportions were positively correlated (Pearson’s *R* = 0.47, *P* < 0.001). Significant overlap was also observed between processing centers for the 8 patients processed at MGH and BIDMC, with higher cross-site reproducibility for Cryo-PRO (*R* = 0.79, *P* < 0.001) than for Ficoll (*R* = 0.38, *P* < 0.001) (Supplemental Figure 8). On a per-patient basis, method and processing center comparisons showed similar patterns, with differences likely reflecting variable T cell recovery (Supplemental Figures 9 and 10).

### Ficoll and Cryo-PRO methods preserve cellular functions required for phagocytosis.

Finally, we sought to measure functional activity in our cryopreserved cells. A key monocyte function is phagocytosis, which requires cells to detect the presence of a pathogen, encapsulate it inside a phagosome, and initiate microbial killing and degradation via fusion with lysosomes and subsequent exposure to hydrolytic enzymes and acidic conditions (33). Detection of phagocytosis therefore indicates coordination of cellular signaling pathways, cytoskeletal rearrangement, and functional organelles. We measured phagocytic activity in PBMCs from samples processed using Ficoll and Cryo-PRO as a means of assessing cell viability, function, and responsiveness to environmental stimuli.

Ficoll and Cryo-PRO samples (1 of each from 9 sepsis patients and 1 healthy participant) were collected, preserved, and frozen. Both sample types then underwent all of the processing steps before flow cytometry sorting, including the red blood cell depletion step for Cryo-PRO samples only (Methods). Cell suspensions were incubated with *E*. *coli* pHrodo bioparticles, which fluoresce in acidic phagolysosomes (34). After incubation, cells were fluorescently stained for viability, CD45, CD15, and CD14, and analyzed with flow cytometry.

**Figure 9:**
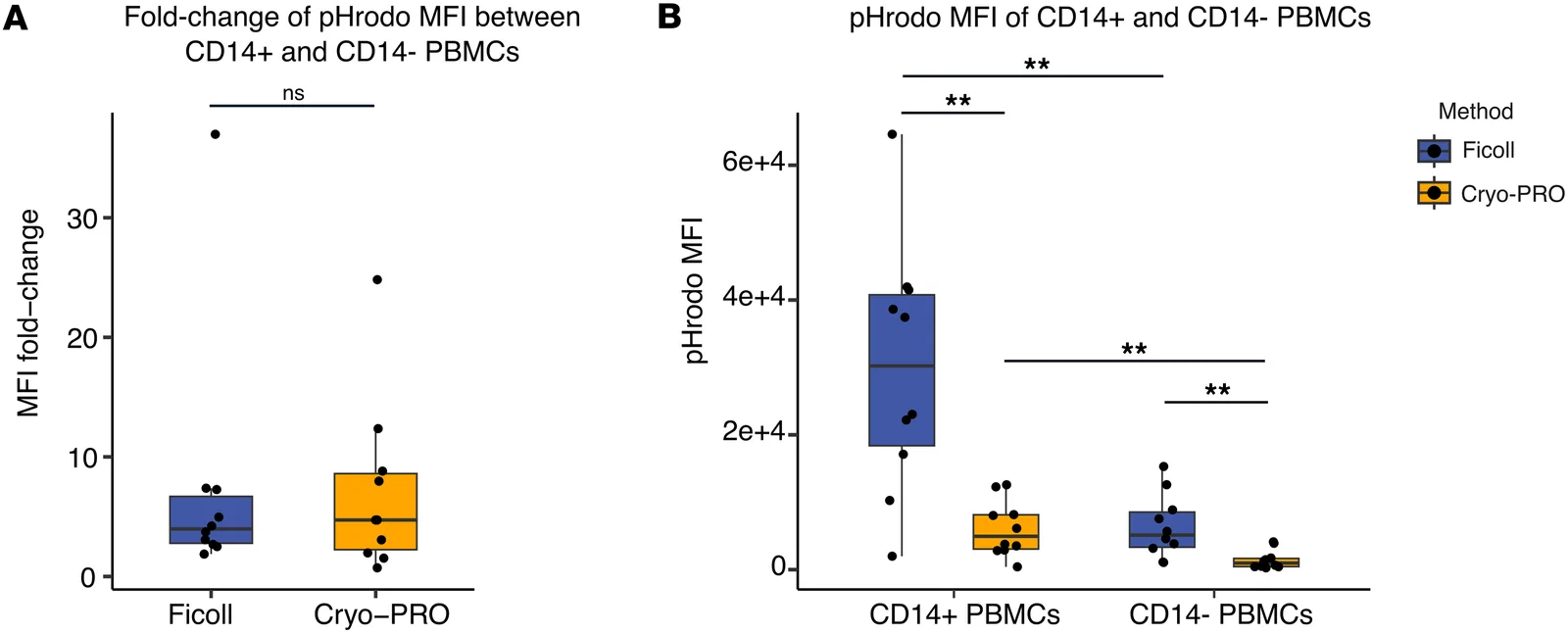
Cryo-PRO preserves phagocytic function in PBMCs. (**A**) Fold-change differences of pHrodo mean fluorescence intensity (MFI) between CD14^+^ and CD14^–^ PBMCs for Ficoll (blue) and Cryo-PRO (yellow) samples. (**B**) Absolute pHrodo MFI of PBMCs, stratified by CD14^+^ expression (horizontal axis) and method (blue = Ficoll, yellow = Cryo-PRO). Box-and-whisker plots indicate the median and IQR (whiskers = 1.5 × IQR). Points represent individual samples. **P < 0.01 by paired, 2-sided *t* test. NS, not significant.

We measured phagocytic activity as the mean fluorescence intensity (MFI) of the pHrodo bioparticles, which emit light at green (FITC) wavelengths. We first confirmed that live PBMCs (DAPI^–^CD45^+^CD15^–^ cells) had greater pHrodo MFI upon addition of the bioparticles (Supplemental Table 4), consistent with engulfment into phagolysosomes. We next stratified the live PBMCs by CD14 expression. Assessment of control wells lacking bioparticles confirmed that fluorescent spillover from the CD14 stain did not impact the pHrodo MFI measurement (Supplemental Table 4). CD14^+^ monocytes are the most abundant phagocytes within PBMCs (35), and within our samples, were expected to show higher pHrodo MFI compared with the CD14^–^ fraction, which consists primarily of lymphocytes with low phagocytic activity, but also includes CD16^+^ monocytes and dendritic cells (36). Accordingly, across all individuals, CD14^+^ PBMCs exhibited higher phagocytic signal than CD14^–^ PBMCs using either processing method (median pHrodo MFI fold change of ~3.98 for Ficoll and ~4.72 for Cryo-PRO), despite substantial inter-individual variability (Figure 9A). The pHrodo MFI of CD14^+^ cells was generally higher in Ficoll than in the corresponding Cryo-PRO sample (median of 30,240 RFU across samples compared with 4,946 RFU across samples) (Figure 9B). Although the reason for this approximately 6-fold change in MFI magnitude by method is uncertain, we hypothesize that it may reflect competition for bioparticles during the incubation before flow cytometry. Due to greater carryover of additional phagocytes (e.g., granulocytes) in Cryo-PRO suspensions, such competition would reduce the effective particle-to–CD14^+^ cell ratio even though CD15^+^ granulocytes are subsequently excluded from analysis by gating. Accordingly, direct between-method comparisons of CD14^+^ pHrodo MFI are confounded due to differences in cellular composition during incubation, whereas the within-method contrast (CD14^+^ vs. CD14^–^) demonstrates that CD14^+^ PBMCs retain detectable phagocytic activity after processing by either method, with comparable increases in bead uptake over the corresponding CD14^–^ cells.

## Discussion

Single-cell transcriptional profiling facilitates high-resolution characterization of circulating immune cells, thereby revealing critical insights into diseases such as sepsis where the immune response plays a pivotal role (37). Performing these investigations with clinical samples is critical for translational goals, as it establishes a direct link between cellular transcriptomics and patient-derived data. However, there are major roadblocks to applying scRNA-seq to clinical blood samples: cost and intensive sample collection that requires more time, and molecular techniques than typically available to clinical study teams. Although scRNA-seq is becoming more economical with dropping sequencing costs and the ability to pool samples, performing scRNA-seq from patient blood still requires the time- and resource-intensive process of Ficoll density gradient centrifugation. This limitation has greatly constrained the application of scRNA-seq in clinical investigations, resulting in smaller patient cohorts from high-resourced settings that may not fully capture the heterogeneity of diseases under study.

Here, we demonstrate that direct cryopreservation of a small volume (~1 mL) of whole blood at the point of care, followed by thawing and PBMC isolation at a centralized research facility, is a viable alternative to on-site Ficoll processing for scRNA-seq, CITE-seq, and TCR repertoire profiling. This simple and streamlined approach greatly reduced the time and technical expertise needed to obtain clinical samples, while still preserving single-cell transcriptomes and surface proteomes in patients with sepsis. We independently identified the same immune cell types and substates in the datasets of Cryo-PRO and Ficoll, including the sepsis-enriched MS1, considered important in sepsis immunopathophysiology (10, 38). We found a high correlation between methods for the abundances of all major cell types and substates. Moreover, patterns of gene and surface protein expression were similar across cell types and substates, with minimal differential gene expression between methods. TCR capture from T cells processed with Cryo-PRO yielded similar clonotype recovery and patterns of clonal expansion to Ficoll. Complementary to our sequencing results, phagocytosis assays demonstrated detectable functional activity in CD14^+^ PBMCs preserved by either method. Together, this substantial equivalence between the gold-standard method of Ficoll processing and Cryo-PRO demonstrates that Cryo-PRO does not introduce major artifacts from processing and generates biologically meaningful results. When deployed across 2 different enrolling emergency departments, Cryo-PRO was robust to variations in collection site and operator, further supporting it as a reliable strategy for expanding multimodal single-cell immune profiling studies.

Recent studies have also demonstrated the compatibility of rapid whole-blood preservation workflows with single-cell profiling (39, 40). Our study extends this literature by providing a paired, patient-matched comparison with standard Ficoll processing in acutely ill patients with sepsis and by demonstrating compatibility with multimodal profiling that includes scRNA-seq, CITE-seq, and scTCR-seq. Prior workflows differ from Cryo-PRO in several practical and technical respects. In one of these studies, a substantial loss in the fractional abundance of myeloid cells was observed when compared with samples obtained using Ficoll (40). Our approach produces closer equivalence with Ficoll across immune cell types and cell substates. Other approaches use cell fixation after thaw to enable scalable profiling (39), which offers logistical advantages but may limit certain downstream applications (41). Technical differences in fixed-cell profiling, namely reliance on prespecified hybridization probes, means that these approaches cannot capture regions of high allelic diversity such as TCR (and B cell receptor) clonotypes (42). Furthermore, fixed-cell kits often exclude capture of mitochondrial reads, limiting the possible quality control comparisons between methodologies. By using unfixed cells, Cryo-PRO remains compatible with a greater suite of profiling options. Other options for rapid sample processing perform cell fixation prior to cryopreservation (43). In addition to the aforementioned limitations of fixed-cell profiling, this approach kills cells and eliminates the option to measure active cellular functions; for example, measuring transcriptional responses to stimuli (44) or Perturb-seq (45). In addition, some of the previous approaches, in part due to smaller sample size, relied on co-clustering of scRNA-seq data with the traditional Ficoll method to assign cell states (39, 40). To be useful at the point of care, a streamlined collection method must stand alone; we therefore independently clustered and analyzed patient-matched data from Cryo-PRO and Ficoll, and observed high technical and biological equivalence.

The Cryo-PRO method has transformative potential for multicenter sample collection and clinical trial enrollment efforts by greatly simplifying on-site protocols for scRNA-seq and transferring the technically demanding steps to a centralized location. The resource demands of on-site processing for scRNA-seq particularly impede studies of highly heterogeneous diseases with acute onset where study collection strategies must be deployable at any time a patient may present. Sepsis is an archetype of such a condition, and sample sizes for scRNA-seq studies of sepsis have, as a consequence, been underpowered to bring the full power of the method to bear on investigating biological reasons underlying the clinical heterogeneity of the condition (10, 12–15). The substantial reduction in technical skill and time requirements for sample processing and preservation (i.e., mean time of 13 minutes for Cryo-PRO vs. 143 minutes for Ficoll) has crucial operational implications in the clinical research setting. Simplifying sample collection also offers an opportunity for improving cost efficiency by enabling the rapid enrollment of many potentially suitable patients for clinical studies, followed by retrospective adjudication to inform the selection of appropriate patients for sequencing. Widening the net of participants enrolled in this manner would better reflect the true patient heterogeneity in conditions under study, enabling post hoc enrichment for rare clinical phenotypes or outcomes. For sepsis, such a strategy could facilitate the derivation of scRNA-seq–based endotypes across large, diverse cohorts, including those from hospitals in underserved communities without dedicated research teams and resources to typically participate in clinical research.

Our study has several limitations. First, our cohort size remains small in the context of sepsis studies. However, to the best of our knowledge, this study (*n* = 24 participants and 32 paired samples) is the largest to date evaluating the feasibility of whole-blood cryopreservation for scRNA-seq, CITE-seq, and TCR profiling in a clinical cohort, and demonstrates substantial equivalence with conventional methods. We aim to further validate Cryo-PRO as a sample processing approach in a larger cohort of participants in the future. Second, although all major cell types and substates had substantial equivalence in patient-level abundance, some cell substate abundances differed between Cryo-PRO and Ficoll methods. High-resolution substate assignment is sensitive to stochasticity from clustering-based methods as well as outlier effects in rare substates, which may explain some of these differences. Unequal cell counts from each sample, which we did not attempt to normalize for beyond setting a minimum threshold for inclusion in correlation calculations (Methods), likely added additional noise to our results. Although cluster-level assignments were done without consideration of aggregate abundance, the analysis was not blinded to method during cell identity assignment, which is a limitation of the study. While some differences between methods may reflect differences in either gene expression or survival by cell type and substate, each method introduces processing steps that may perturb transcription, i.e., centrifugation for 2 hours through a density gradient followed by exposure to cryopreservatives, freezing, thawing, and flow cytometry for Ficoll; or exposure to DMSO, freezing, thawing, magnetic cell separation, and flow cytometry for Cryo-PRO. Additionally, 8 participants were processed in parallel at 2 sites, with a resulting mean delay of just over 2 hours prior to processing by either method, which may have affected subsequent sequencing results in these samples. Despite these limitations, the overall agreement in cell type and substate proportions between methods, and the minimal perturbations seen in differential expression by method, suggest that major transcriptional signals that reflect relevant biology are preserved. Third, we evaluated one function (i.e., phagocytosis) of the PBMCs isolated by Cryo-PRO; additional assays will be needed to further assess the functional capacity of PBMCs isolated using the Cryo-PRO method. Although the absolute magnitude of phagocytic activity differed between methods, the within-method contrast between CD14^+^ and CD14^–^ PBMCs suggests that detectable phagocytic activity was preserved with each method. We expect that the preservation of relative biological function by cell state demonstrates potential for Cryo-PRO cells to be used in in vitro assays and present these findings as a basis for further experimentation. Future studies are needed to fully assess functional activity of cells obtained using Cryo-PRO.

Finally, a major limitation to both Ficoll and Cryo-PRO methods is their failure to preserve neutrophils, which are eliminated in the density gradient separation step (Ficoll) or rupture during freeze/thaw (Cryo-PRO). For these reasons, preserving neutrophils for scRNA-seq remains a large challenge despite their importance. Neutrophils play critical roles in sepsis pathophysiology and dominate bulk sequencing transcriptional signatures in sepsis and many other critical illnesses; these methods therefore only capture the response of PBMCs in sepsis and not the full circulating immune response.

By simplifying sample collection at the point of care, Cryo-PRO can unlock greater potential of scRNA-seq to study the biology of complex clinical conditions across multiple collection sites, including lower-resource settings, thus improving capture of true disease heterogeneity. This method greatly lowers the barrier to embedding scRNA-seq in randomized clinical trials, which would enable post hoc analyses to identify endotypes that may selectively respond to therapeutic interventions. In addition, Cryo-PRO could enhance the cost efficiency of scRNA-seq by minimizing labor and costs of “overcollection” at the point of care, reserving PBMC isolation and scRNA-seq only for samples from patients who display clinical phenotypes or disease trajectories of interest on subsequent adjudication. Thus, Cryo-PRO has substantial potential to expand the application of scRNA-seq towards personalized medicine in complex and heterogeneous conditions like sepsis, and this work represents an important step towards that goal.

## Methods

### Sex as a biological variable.

We included 8 female patients and 15 male patients in our study, in addition to one male healthy control participant. To account for patient heterogeneity in our study, including potential effects of sex as a biological variable, all comparisons in our study were made by comparing samples processed with different methods but collected from the same individual.

### Patient enrollment and clinical adjudication.

This study was conducted at MGH and BIDMC. Inclusion criteria were adult patients arriving at the Emergency Department with evidence of organ dysfunction for whom bacterial infection was possible or suspected. Eligible patients had a blood sample collected under an IRB-approved alteration of informed consent, which allowed a research sample to be drawn simultaneously with the initial clinical blood draw. Informed consent was obtained from the patient or a surrogate at a later time after initial resuscitation.

Samples were collected for 100 patients during a 12-month period from April 2023 to March 2024. Of these, 84 provided consent for research use and were considered enrolled; samples from patients who did not provide consent were discarded. Clinical data were collected on all enrolled participants and entered into REDCap by clinical research coordinators. Physician adjudication was later performed via retrospective chart review with access to all available clinical data and notes during the individual’s hospitalization. Participants were adjudicated as meeting Sepsis-3 criteria (18) for sepsis or septic shock during the first 48 hours of hospitalization, or as having infection without sepsis versus a non-infectious cause of illness. For the current analysis, we prioritized sequencing in those participants adjudicated as sepsis and septic shock, and selected 23 for sequencing.

### Sample collection at clinical sites.

Research blood samples were collected in 10-mL EDTA tubes. Up to 20 mL was collected if patient samples were being parallel-processed at both clinical sites; up to 10 mL was collected if patient samples were being processed at only a single site. For samples parallel-processed at both clinical sites, 1 of the two 10-mL tubes collected at the enrolling site was couriered to the second site. This resulted in a delay in processing of about 2 hours on average; samples from one participant were delayed more than 3 hours. Samples obtained for single-site processing were taken directly to the on-site lab for immediate processing. All 10-mL samples underwent cryopreservation of whole blood (2 mL) and on-site density gradient centrifugation with Ficoll (~3 to 6 mL) as described below. Up to 3 mL of the collected whole-blood sample was used for other research purposes.

### On-site whole-blood cryopreservation for Cryo-PRO.

For immediate whole-blood cryopreservation, 2 mL of blood was mixed with 200 μL DMSO (final 10% v/v). Two 1-mL aliquots in cryovials were then prepared per sample and were slowly cooled using a Mr Frosty (Sigma-Aldrich) at –80°C. Aliquots were stored onsite at –80°C for less than 1 month before being transported to the Broad Institute on dry ice and immediately stored at –140°C until the time of sequencing. Duplicate aliquots served as backups.

### On-site density gradient centrifugation (Ficoll) and cryopreservation of PBMCs.

Density gradient centrifugation was performed on the remaining blood in the EDTA tubes after cryopreservation aliquots had been obtained (~3 to 6 mL). Blood with EDTA was diluted 1:1 with room temperature PBS and layered over Ficoll-Paque PLUS density gradient media (Cytivia) in SepMate tubes (STEMCELL Technologies), then centrifuged at 1,200*g* for 20 minutes at 20°C with slow acceleration and the brake off. The buffy coat layer was collected and washed twice with cold RPMI (Gibco) before cells were counted, resuspended in CryoStor CS10 (STEMCELL Technologies), and aliquoted into cryotubes, targeting 1 million cells per vial. Samples were cooled, stored, and transported in the same manner as the Cryo-PRO samples.

### Healthy donor blood cryostorage.

Fresh healthy donor blood in EDTA tubes was obtained from Research Blood Components and processed within 2 hours of receipt. Whole-blood Cryo-PRO and Ficoll PBMC cryostorage steps were performed as above, although all processing steps occurred at the Broad Institute.

### Presequencing processing for both Ficoll and Cryo-PRO samples.

All subsequent processing and analysis steps were performed at the Broad Institute. On the day of flow cytometry sorting and 10X Genomics Chromium processing, a sample of cryopreserved whole blood (for Cryo-PRO) and a Ficoll sample were thawed for each patient. Sequencing batches were designed to contain 4 Ficoll samples and 4 patient-matched Cryo-PRO samples to minimize the effect of sequencing batch variation on the method comparison; therefore, 8 samples total were processed in parallel.

For each of the 4 Cryo-PRO samples, 1 mL of cryopreserved whole blood was thawed in a 37°C water bath for 1 minute, 15 seconds and transferred into a 5-mL polystyrene round-bottom tube using 1 mL of PBS containing 2 mM EDTA and 2.5% FBS. Samples were immediately depleted of red blood cells using the STEMCELL Technologies EasySep RBC depletion kit. Briefly, the diluted blood was mixed with 50 μL of the RBC depletion reagent before immediately being placed on a magnet for 5 minutes at room temperature. The supernatant was pipetted off and mixed with an additional 50 μL of RBC depletion reagent in a new tube before another immediate 5-minute magnet incubation. At the end of the second incubation, the supernatant was transferred into 8.5 mL of FBS-RPMI (RPMI + 10% FBS + 1× penicillin/streptomycin) on ice. These steps were performed in parallel for the 4 Cryo-PRO samples. We did not systematically evaluate any alternative methods of sample purification.

For each of the 4 Ficoll samples, 1 vial per patient was thawed in a 37°C water bath for 1 minute, 15 seconds before transfer with 1 mL of FBS-RPMI into 8.5 mL of FBS-RPMI on ice. For patients with 3 or more Ficoll vials, 2 vials were thawed and combined to improve cell recovery. These steps were performed in parallel for the 4 Ficoll samples.

For the subsequent steps, Cryo-PRO and Ficoll samples were processed identically and in parallel. All samples were centrifuged to pellet the cells (300*g*, 5 minutes, 4°C), then resuspended with FACS-PBS (PBS + 2 mM EDTA + 2.5% FBS) and centrifuged again. Each sample was then resuspended in 50 μL FACS-PBS and incubated on ice with a hashtag oligo for pooled sequencing (TotalSeq anti-human Hashtags, BioLegend), an Fc receptor blocking solution (Human TruStain FcX, BioLegend), and flow cytometry stains (DAPI solution,Thermo Fisher Scientific; Alexa Fluor 700 anti-human CD15 [clone HI98], BioLegend; FITC anti-human CD235a [clone HI264], BioLegend; and PE anti-human CD45 [clone HI30], BioLegend). Samples were then washed in cold FACS-PBS and sorted on a SONY MA800 cell sorter to select DAPI^–^CD15^–^CD235a^–^CD45^+^ cells, targeting 50,000 cells per sample.

After sorting, the hashed cells from all 8 samples were pooled, pelleted (300*g*, 5 minutes, 4°C in FACS-PBS), and resuspended in a CITE-seq cocktail for surface proteome measurement (19) for a final incubation on ice. After 20 minutes, the cells were washed twice more (centrifugation at 300*g*, 5 minutes, 4°C followed by resuspension in PBS + 2.5% FBS), counted, and resuspended in PBS plus 2.5% FBS for a target concentration of 1,000 cells/μL.

### PBMC viability measurements.

Data collected during the flow cytometry sorting step (see *Presequencing processing for both Ficoll and Cryo-PRO samples* above) were used to assign a PBMC viability percentage to each sample (Figure 2). Singlet cells staining as CD45^+^CD235a^–^CD15^–^ were gated on the live/dead stain DAPI to calculate the proportion of viable (DAPI^–^) cells. For each method, only live (DAPI^–^) cells were carried forward for library construction.

### Library construction and scRNA-seq.

Droplet-based single-cell RNA capture and RNA and ADT library construction was performed with the Chromium single-cell 5′ kit v2 (10X Genomics, Inc). Forty microliters of cells was loaded onto the Chromium Chip K, and Gel Bead-in Emulsion creation and library construction followed according to the manufacturer’s protocol (46). Eight batches of libraries were prepared (including gene expression libraries and cell surface protein libraries), with each batch barcoded using the 10X Dual Index Kit and sequenced altogether. Gene expression libraries were initially sequenced at low depth (~200 reads/cell) using the Illumina MiniSeq 150 Cycle Hi-Output Kit for a quality check and cell count estimate to inform library balancing. Rebalanced libraries targeting 50,000 reads/cell for gene expression and 10,000 reads/cell for surface proteins were then sequenced on an Illumina NovaSeq S4.

### Data preprocessing.

FASTQ files were aligned to a reference genome (GRCh38) using the Cell Ranger v6 pipeline by 10X Genomics. Demultiplexing and multiplet detection with patient hashtag oligos was performed using the Cumulus pipeline (47). Filtered gene expression matrices and CITE-seq matrices were then analyzed using the Seurat V5 package in R. Multiplets and cell barcodes without corresponding gene expression, CITE-seq, and demultiplexing data were removed. Genes present in less than 10 cells were removed. Sequencing data from each method was split into 2 datasets and analyzed independently. For each set, RNA expression data were normalized, scaled, and integrated between sequencing batches using the top 2,000 most variable genes. Scaled CITE-seq data were integrated by finding multimodal neighbors using the first 50 principal components of scRNA-seq and CITE-seq data.

### Clustering and substate identification.

Clustering was performed using the resulting weighted-nearest-neighbors graph, and the Clustree package (48) was used to determine clustering resolution. Cell types were assigned to clusters using top marker genes for each cluster (determined by the Wilcoxon rank-sum test, Bonferroni-corrected *P* < 0.05, ranked by fold change), and cell substates were assigned using top marker genes obtained by subsetting and reclustering cells from each cell type at a higher resolution. Classification of cell types and substates was cross-referenced using the annotated Azimuth reference dataset (20). Clusters were defined as low quality if over 20% of cells in the cluster were cells with mitochondrial genes representing 10% or more of total genes detected in that cell. Low-quality clusters were removed from further analysis as part of an extended quality control. After method-independent cell substate assignment, the Ficoll and Cryo-PRO datasets were combined and a UMAP was generated using the weighted-nearest-neighbors graph for the purpose of data visualization.

### Differential gene expression.

Statistical analysis of differentially expressed genes was performed using the DESeq2 package, as described below in *Statistics*.

### Cell type and substate abundance.

PBMC cell type proportions were calculated as a fraction of all major cell types (monocytes, B cells, T cells, natural killer cells, and dendritic cells). Cell substate proportions were calculated as a fraction within the parent cell type. Cell clusters defined as low quality, or belonging to a class of cells other than PBMCs (e.g., platelets and hematopoietic stem and progenitor cells), were not included in proportion calculations. Samples with fewer than 1,000 total cells were not included in correlation calculations (Figures 6 and 7, and Supplemental Figures 4 and 6). When possible, the Ficoll:Cryo-PRO comparisons were made using samples processed at the same site at which they were collected. In the case of participant 17, one sample yielded less than 1,000 cells, so the Ficoll:Cryo-PRO samples processed at the alternative site were used in calculations instead. Pearson correlations (*R*) were calculated and reported for scatter plots of cell types and substates.

### scTCR-seq and repertoire analysis.

Paired α/β TCR sequences were obtained using the 10X Genomics 5′ V(D)J Immune Profiling workflow (46). Following single-cell capture and cDNA amplification, TCR libraries were constructed in parallel with gene expression libraries from the same droplets, according to the manufacturer’s protocol. Libraries were sequenced to sufficient depth to recover full-length V(D)J transcripts. TCR reads were processed using Cell Ranger pipelines to assemble CDR3 sequences for both TCRα and TCRβ chains. Cells lacking a productive TCR sequence were excluded. Productive paired TCRαβ chains were extracted using the combineTCR() function in the scRepertoire R package (49). Clonotypes were defined by identical CDR3 amino acid sequences for both α and β chains, and clonal expansion was visualized using scRepertoire functions.

### Phagocytosis assays.

Samples were collected and stored as described above. One Ficoll and one Cryo-PRO sample from 10 participants (9 patients, 1 healthy individual) were used. Due to limited samples per patient, technical replicates were not possible. Sample thaw followed steps in presequencing processing through the first wash in FACS-PBS. Each sample was then split into 2; one aliquot of each sample was incubated with reconstituted pHrodo Green *E*. *coli* BioParticles Conjugate for Phagocytosis (Invitrogen) for 10 minutes at 37°C; the other aliquot underwent identical handling without particles (control). After 10 minutes, samples were washed according to the manufacturer’s protocol and stained for flow cytometry. Cells were stained using APC–anti-human CD15 (clone HI98), Spark PLUS UV395–anti-human CD14 (clone S18004B), PE–anti-human CD45 (clone HI30) (BioLegend), and DAPI (Invitrogen), and then incubated on ice for 20 minutes. Cells were then washed in cold FACS-PBS, resuspended in 200 μL of FACS-PBS, and analyzed for surface markers using a Beckman CytoFLEX LX flow cytometer. Phagocytosis was measured simultaneously using the FITC channel. Data were analyzed using FlowJo to identify and stratify cell populations of interest (CD14^+^ PBMCs and CD14^–^ PBMCs), and to quantify phagocytosis using FITC MFI. Potential confounding effects of spectral spillover were minimized using a compensation matrix at the time of acquisition. Additionally, all reported FITC MFI values of cell populations with bioparticles were normalized by subtracting the FITC MFI of the corresponding population in the no-particle control wells to account for spillover into the FITC channel.

### Figure generation.

Figures 2–9 and all supplemental figures were generated in R using the ggplot2 package, the scCustomize package (50), the Seurat package (20), and the scRepertoire package (49).

### Statistics.

Statistical significance values (*P* values) for Figure 2, A and B, and Figure 9, A and B, were generated using a paired, 2-sided *t* test. The *P* value for Supplemental Figure 1 was generated using a Welch’s 2-sample *t* test of unequal variance. To assess differential gene expression between methods, scRNA-seq data were first pseudobulked by sample, generating 1 pseudobulk profile per sample for each method comparison, to minimize *P* value inflation (51), and FindMarkers with the DESeq2 package was used to detect differentially expressed genes. The same approach was applied within cell types by pseudobulking cells by sample × cell type. Unless otherwise stated, tests were 2-sided. For differential gene expression analyses, *P* values were adjusted for multiple testing using the Benjamini-Hochberg false discovery rate; we report adjusted *P* values. For marker-gene discovery, genes were included if log_2_(fold change) was greater than 0.25, the genes were expressed in over 25% of cells in the cluster, and the Bonferroni-corrected *P* value was less than 0.05. For correlation analyses, we report Pearson’s *R*, with statistical significance assessed via 2-sided *t* test.

### Study approval.

This study was approved by the Massachusetts General Brigham IRB (2022P002833). Research blood was collected under an IRB-approved alteration of informed consent; written informed consent was subsequently obtained from participants or legally authorized representatives.

### Data availability.

scRNA-seq data are available on the Broad Institute Single Cell Portal (accession: https://singlecell.broadinstitute.org/single_cell/study/SCP3740/a-whole-blood-cryopreservation-method-streamlines-clinical-sample-collection-for-multimodal-single-cell-immune-profiling-in-sepsis#study-visualize). Data from scRNA-seq analysis and all other data shown can be accessed in the Supporting Data Values file.

## Author contributions

MRF, NIS, and RPB conceived the study. MRF and NIS supervised enrollment and sample collection and performed clinical adjudications. ACC, RH, OKN, CAZ, MKD, SM, AM, and BAP consented and enrolled patients and performed on-site processing steps. MRF, RPB, POA, and AKD designed the experiments. AKD conducted experiments. AKD and POA performed data analysis. AKD, POA, MRF, and RPB prepared the manuscript. AKD and POA contributed equally to the manuscript; authorship order was assigned by duration of involvement in the project.

## Conflict of interest

AKD, POA, MRF, and RPB are inventors on a pending U.S. non-provisional patent application, assigned to the Broad Institute, Inc. and The General Hospital Corporation, related to certain subject matter described in this manuscript, and published as US 2026/0078428.

## Funding support

This work is the result of NIH funding, in whole or in part, and is subject to the NIH Public Access Policy. Through acceptance of this federal funding, the NIH has been given a right to make the work publicly available in PubMed Central.

- NIH grants R21GM148826 and R01AI153142.

## Supplementary Material

Supplemental data

Supplemental tables 1-4

Supporting data values

## Version 1 07/14/2026

In-Press Preview

## Version 2 08/24/2026

Electronic publication
