# Supplementary material for: A whole-blood cryopreservation method streamlines clinical sample collection for multimodal single-cell immune profiling in sepsis
Source: JCI Insight. 2026 Jul 14;11(16):e204610. doi: 10.1172/jci.insight.204610 (PMC13502171; doi:10.1172/jci.insight.204610)
Supplement: Supplementary file 1 [file jciinsight-11-204610-s146.pdf]

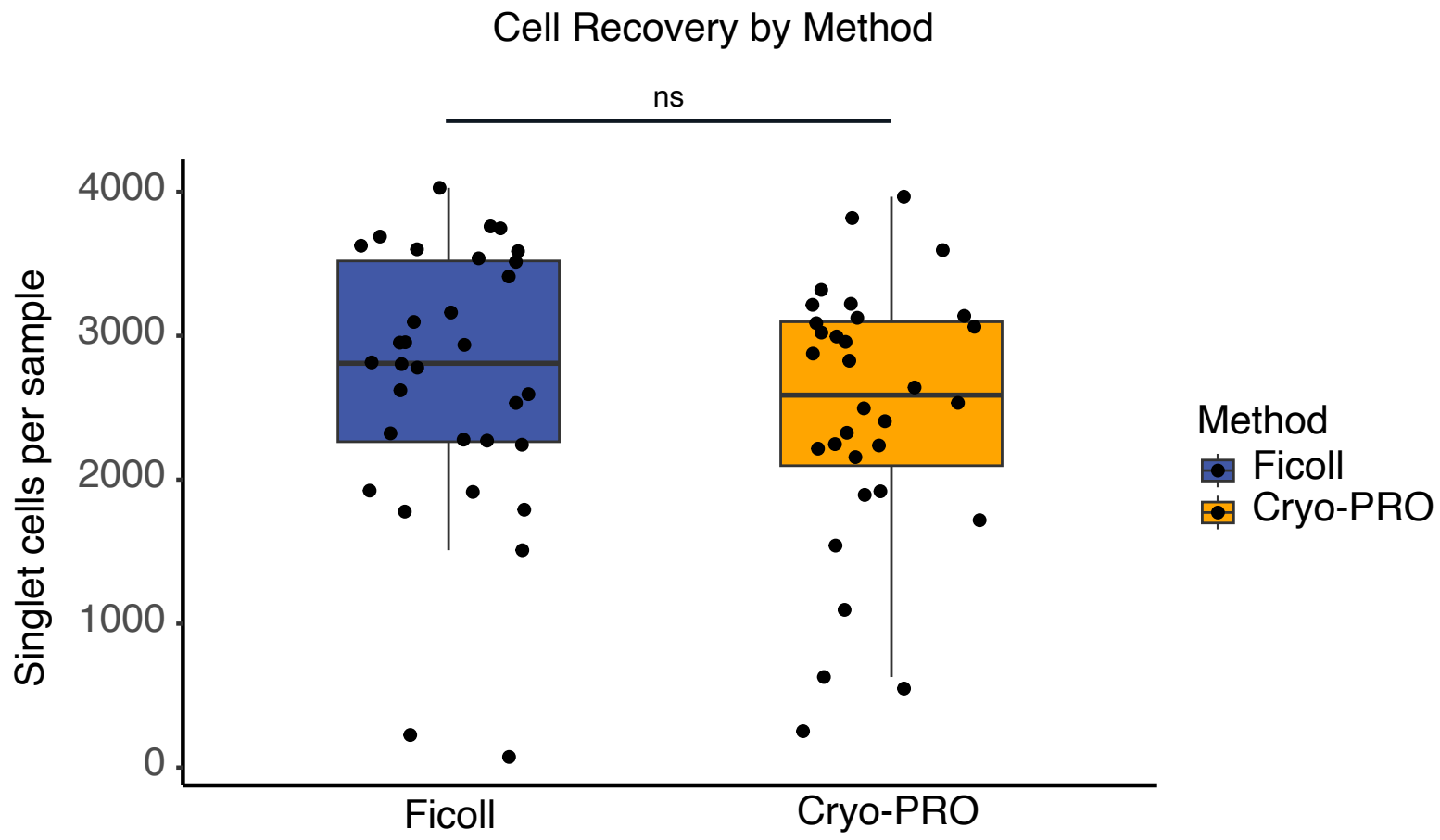

Supplemental Figure 1. Number of singlet cells sequenced per method. Starting blood sample volume was variable in Ficoll samples and was 1 mL in Cryo-PRO samples. Because Ficoll input volumes varied and were not recorded precisely, these values are not a direct comparison of recovery efficiency between methods. Statistical significance was generated using a Welch's two-sample t test assuming unequal variances (*p < 0.05, **p < 0.01, ***p < 0.001, ns = not significant).

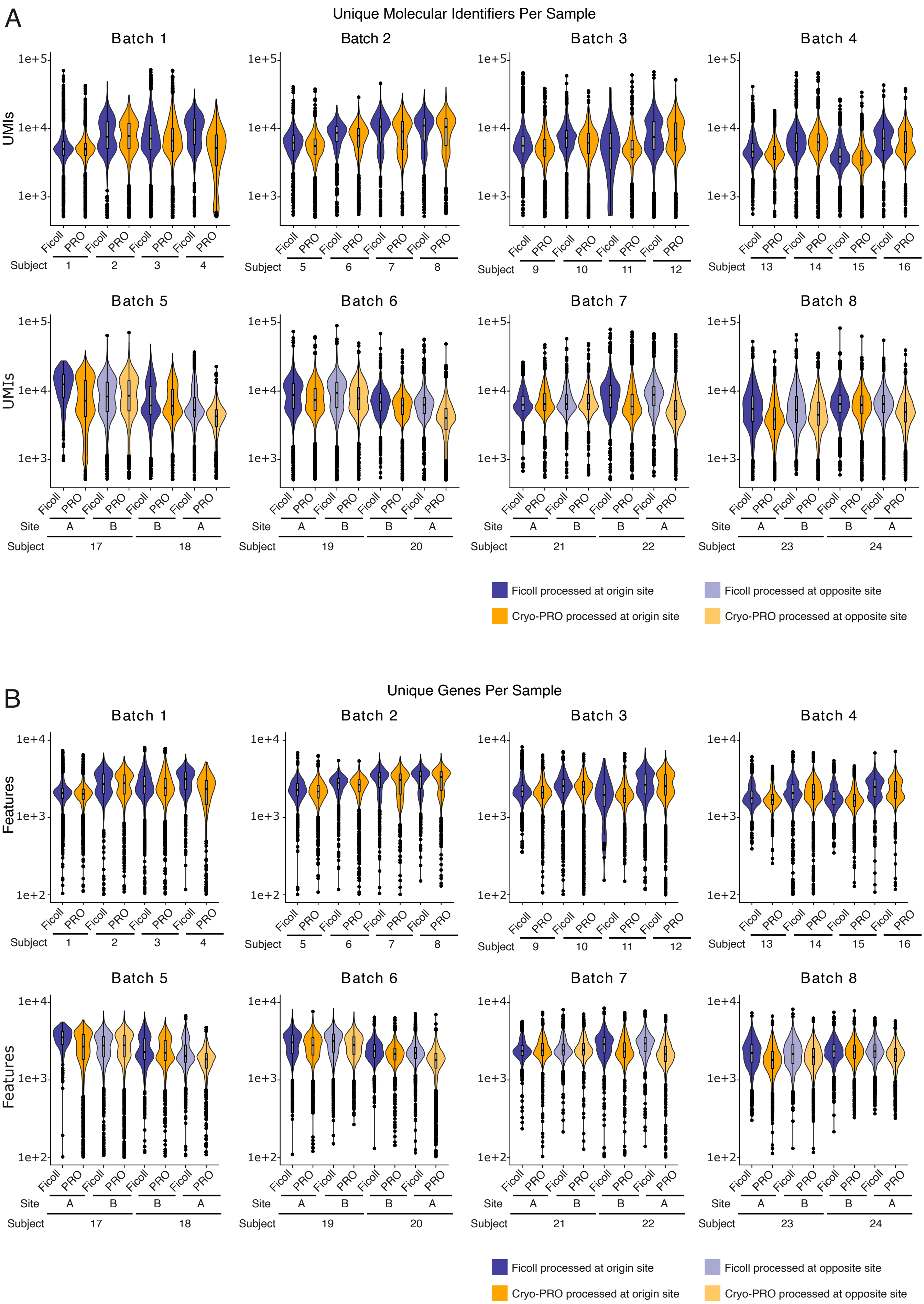
A
Unique Molecular Identifiers Per Sample
Batch 1
Batch 2
Batch 3
Batch 4
Batch 5
Batch 6
Batch 7
Batch 8
UMIs
1e+5
1e+4
1e+3
Ficoll
PRO
Subject
Site
A
B
1
2
3
4
5
6
7
8
9
10
11
12
13
14
15
16
17
18
19
20
21
22
23
24
Ficoll processed at origin site
Ficoll processed at opposite site
Cryo-PRO processed at origin site
Cryo-PRO processed at opposite site
B
Unique Genes Per Sample
Features
1e+4
1e+3
1e+2

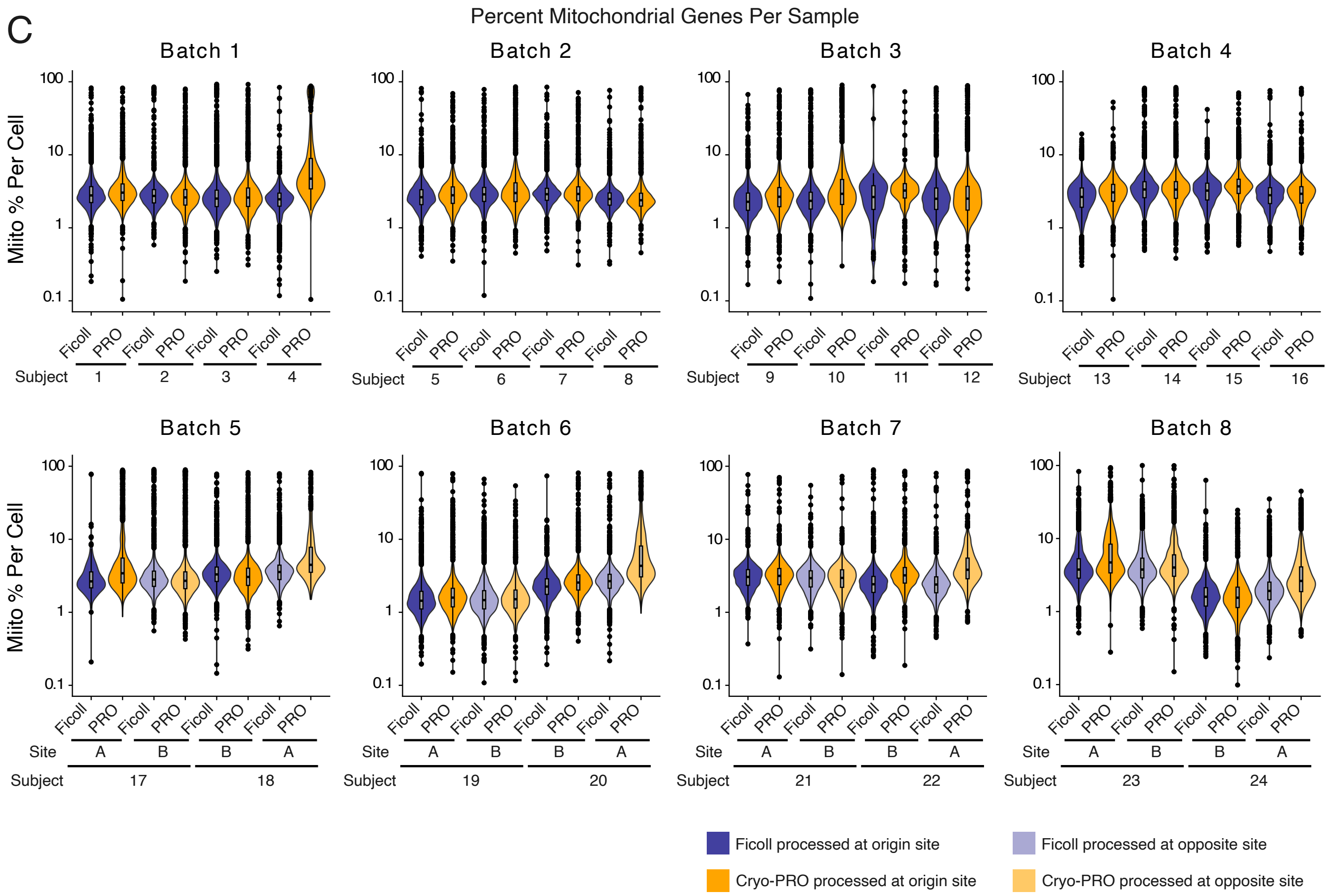
C
Percent Mitochondrial Genes Per Sample
Batch 1
Batch 2
Batch 3
Batch 4
Batch 5
Batch 6
Batch 7
Batch 8
Mito % Per Cell
100
10
1
0.1
Ficoll
PRO
Subject
Site
A
B
Ficoll processed at origin site
Ficoll processed at opposite site
Cryo-PRO processed at origin site
Cryo-PRO processed at opposite site

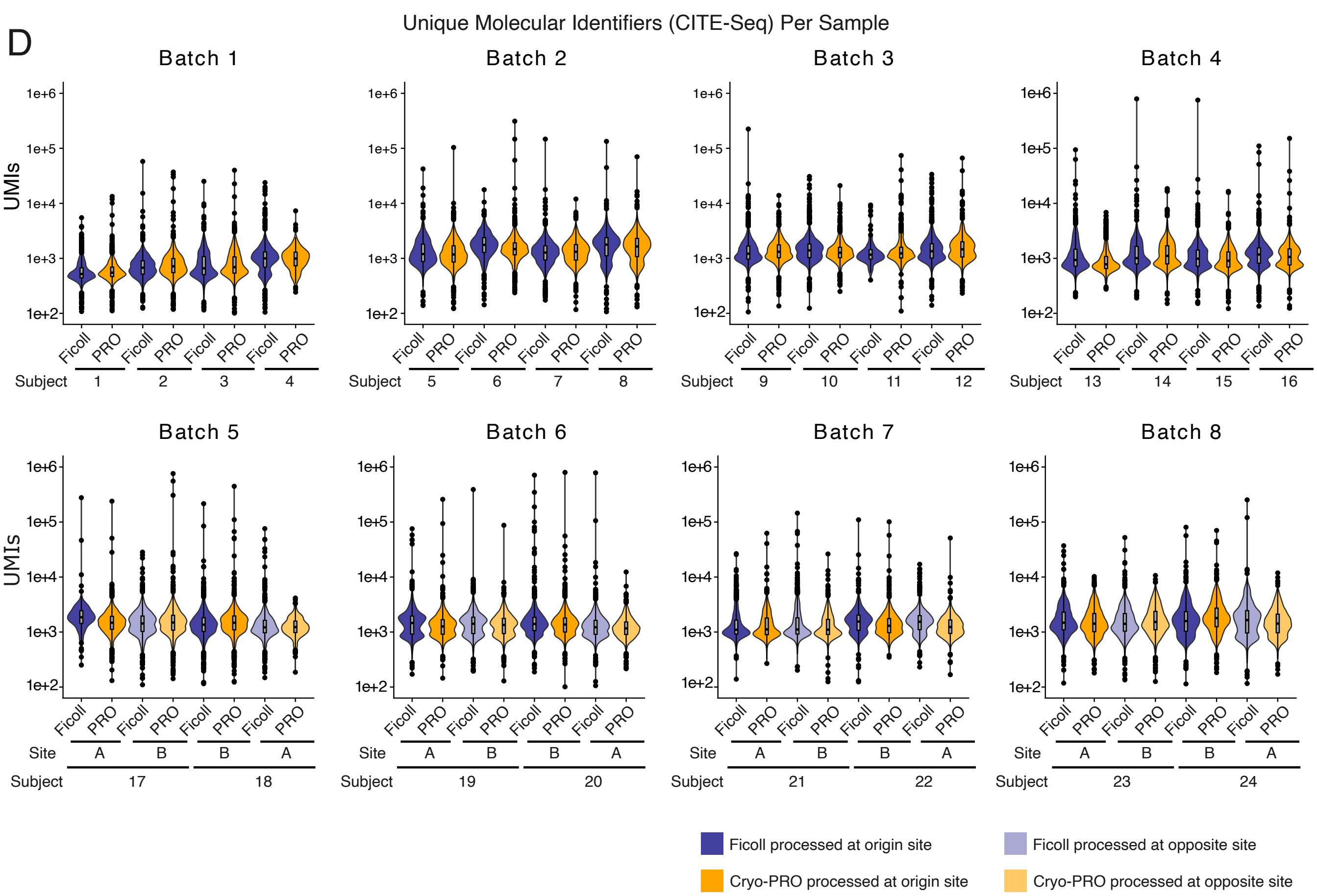
D
Unique Molecular Identifiers (CITE-Seq) Per Sample
Batch 1
Batch 2
Batch 3
Batch 4
Batch 5
Batch 6
Batch 7
Batch 8
UMIs
1e+6
1e+5
1e+4
1e+3
1e+2
Ficoll
PRO
Subject
Site
A
B
Ficoll processed at origin site
Ficoll processed at opposite site
Cryo-PRO processed at origin site
Cryo-PRO processed at opposite site

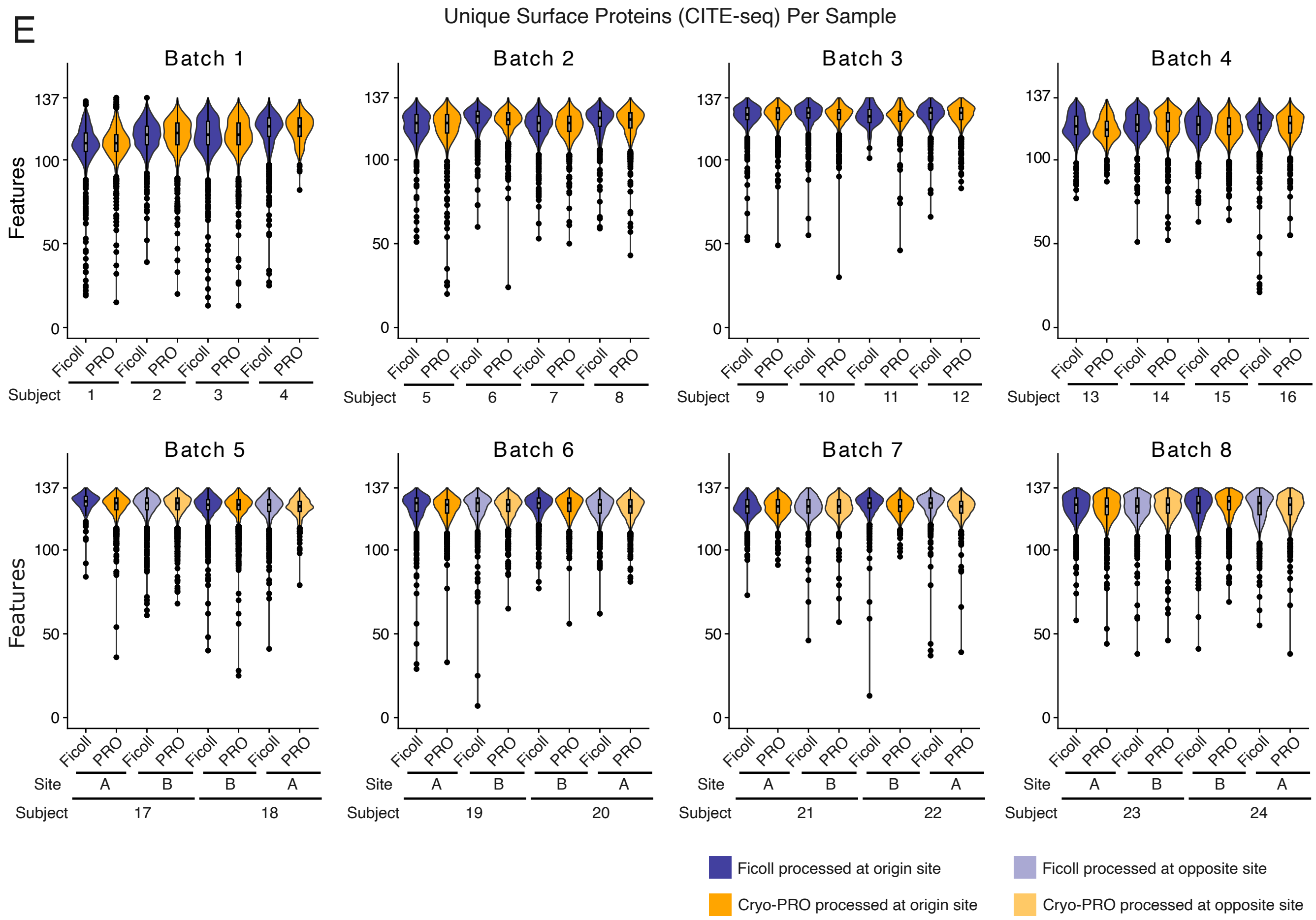

Supplemental Figure 2. Per-sample distributions of **(A)** UMIs of RNA transcripts, **(B)** unique genes, **(C)** percentage of mitochondrial transcripts, **(D)** unique surface protein features detected via CITE-seq, and **(E)** UMIs of surface protein features detected via CITE-seq per cell. Batches represent samples that were thawed, processed and sequenced together. Ficoll and Cryo-PRO samples from the same patient are plotted next to each other. For patients where parallel processing occurred at both clinical sites (bottom rows), the samples processed at the opposite site of enrollment are shown in lighter shades. A total of 137 different surface proteins were queried in the CITE-seq analysis. Violin plots show the distribution of each metric detected per cell; embedded boxplots indicate the median and IQR (whiskers = 1.5x IQR). PRO denotes Cryo-PRO.

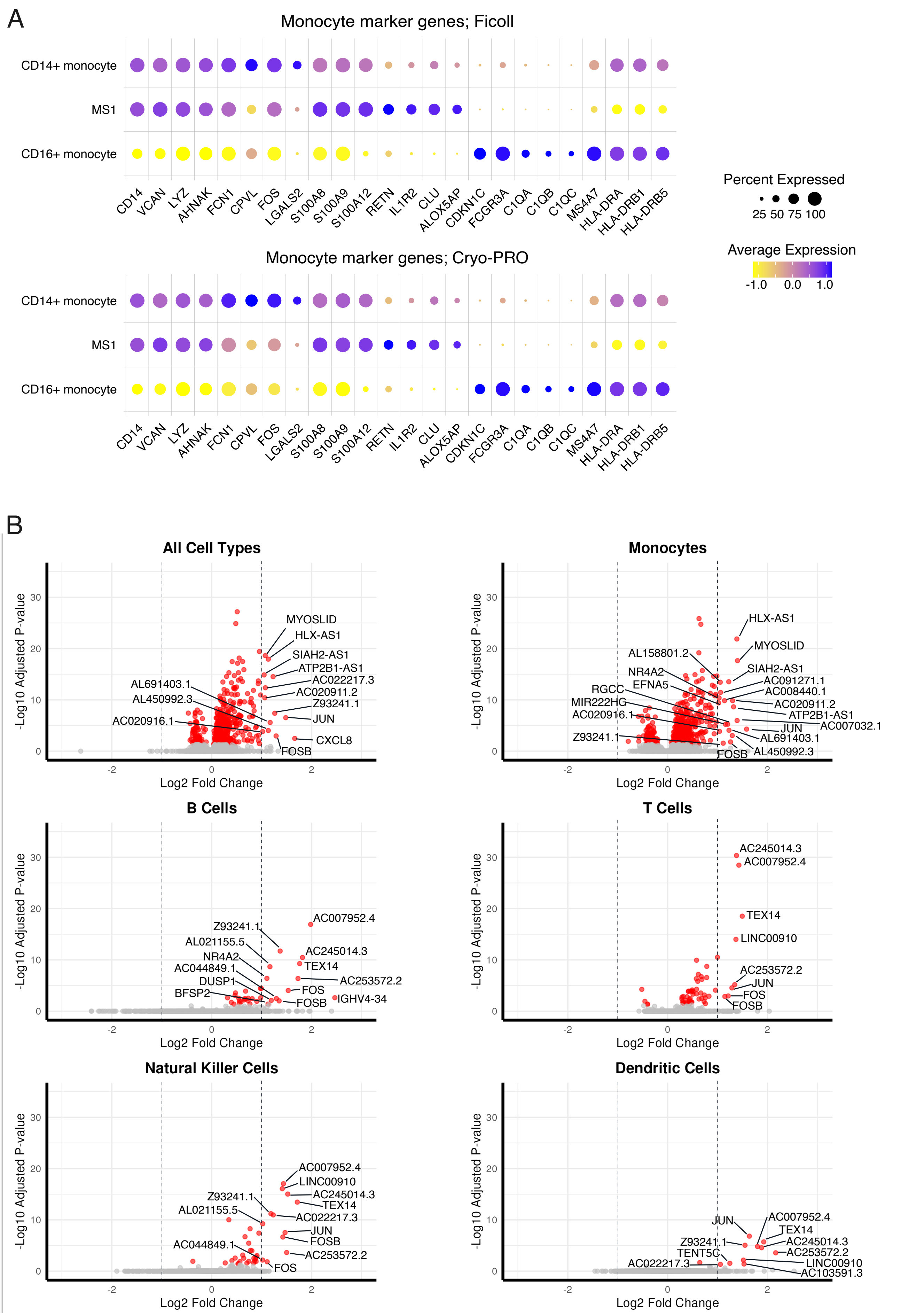
A
Monocyte marker genes; Ficoll
CD14+ monocyte
MS1
CD16+ monocyte
CD14
VCAN
LYZ
AHNAK
FCN1
CPVL
FOS
LGALS2
S100A8
S100A9
S100A12
RETN
IL1R2
CLU
ALOX5AP
CDKN1C
FCGR3A
C1QA
C1QB
C1QC
MS4A7
HLA-DRA
HLA-DRB1
HLA-DRB5
Percent Expressed
25 50 75 100
Average Expression
-1.0 0.0 1.0
Monocyte marker genes; Cryo-PRO
B
All Cell Types
Monocytes
B Cells
T Cells
Natural Killer Cells
Dendritic Cells
-Log10 Adjusted P-value
Log2 Fold Change
MYOSLID
HLX-AS1
SIAH2-AS1
ATP2B1-AS1
AC022217.3
AC020911.2
Z93241.1
JUN
CXCL8
FOSB
AL691403.1
AL450992.3
AC020916.1
AL158801.2
NR4A2
EFNA5
RGCC
MIR222HG
AC091271.1
AC008440.1
AC007032.1
AC007952.4
AL021155.5
AC044849.1
DUSP1
BFSP2
AC245014.3
TEX14
AC253572.2
FOS
IGHV4-34
LINC00910
TENT5C
AC103591.3

Supplemental Figure 3. Dot plots of marker gene expression by each monocyte substate **(A)**. Color represents scaled relative expression (blue = higher expression). Size represents the proportion of cells in each substate where the feature was detected. Volcano plots showing genes differentially up-regulated (positive Log2FC) or down-regulated (negative Log2FC) in Ficoll compared to Cryo-PRO after pseudobulk analysis **(B)**. Genes with adjusted p-values of less than 0.05 are shown in red; those with $p < 0.05$ and abs(log2FC) > 1 are labeled. Plots are shown for differential gene expression among all cells (top left) and for each major cell type (subsequent plots).

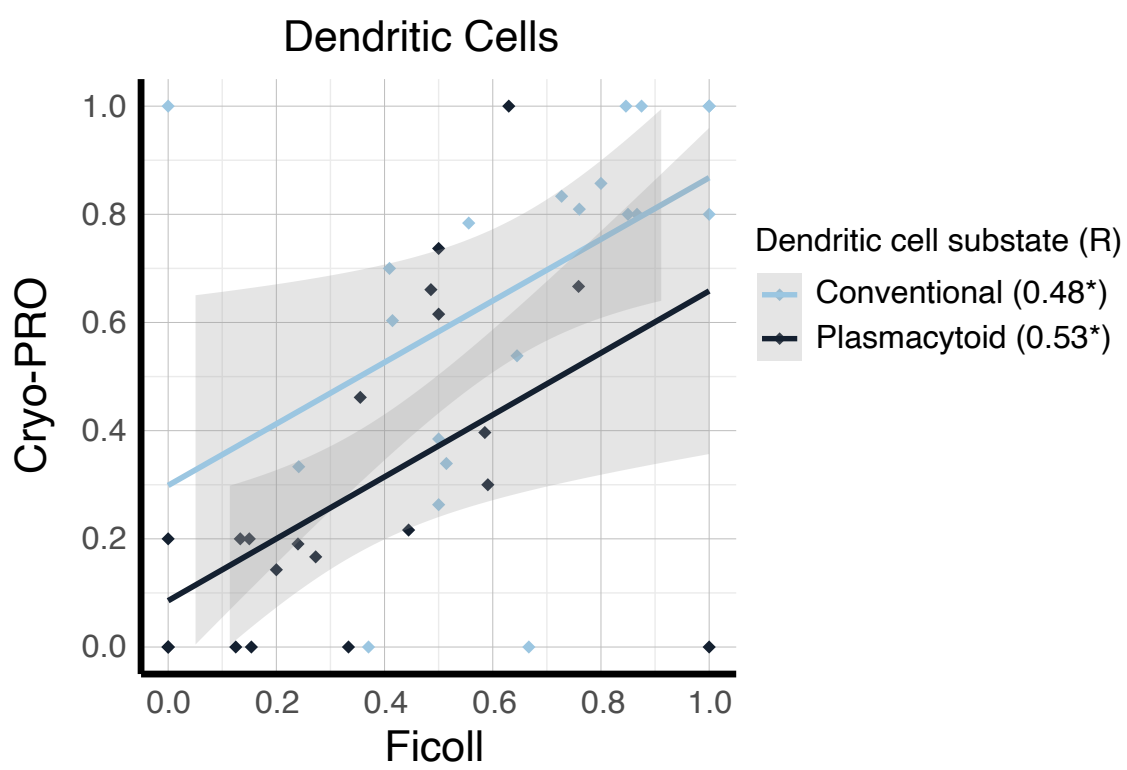

Supplemental Figure 4. Scatter plot of dendritic cell substate proportion from Ficoll and Cryo-PRO. Each point represents the proportion of one cell substate from one patient sample, as measured by each method. Each cell substate is represented by a different color. Proportion is the number of cells of one cell substate divided by the total number of dendritic cells from that patient sample. Patient-paired Ficoll:Cryo-PRO samples are plotted to assess correlation in method for each patient. Trendlines indicate a linear regression fit with shaded 95% confidence intervals. Pearson's correlations (R) are shown for all correlations (* $p < 0.05$, ** $p < 0.01$, *** $p < 0.001$).

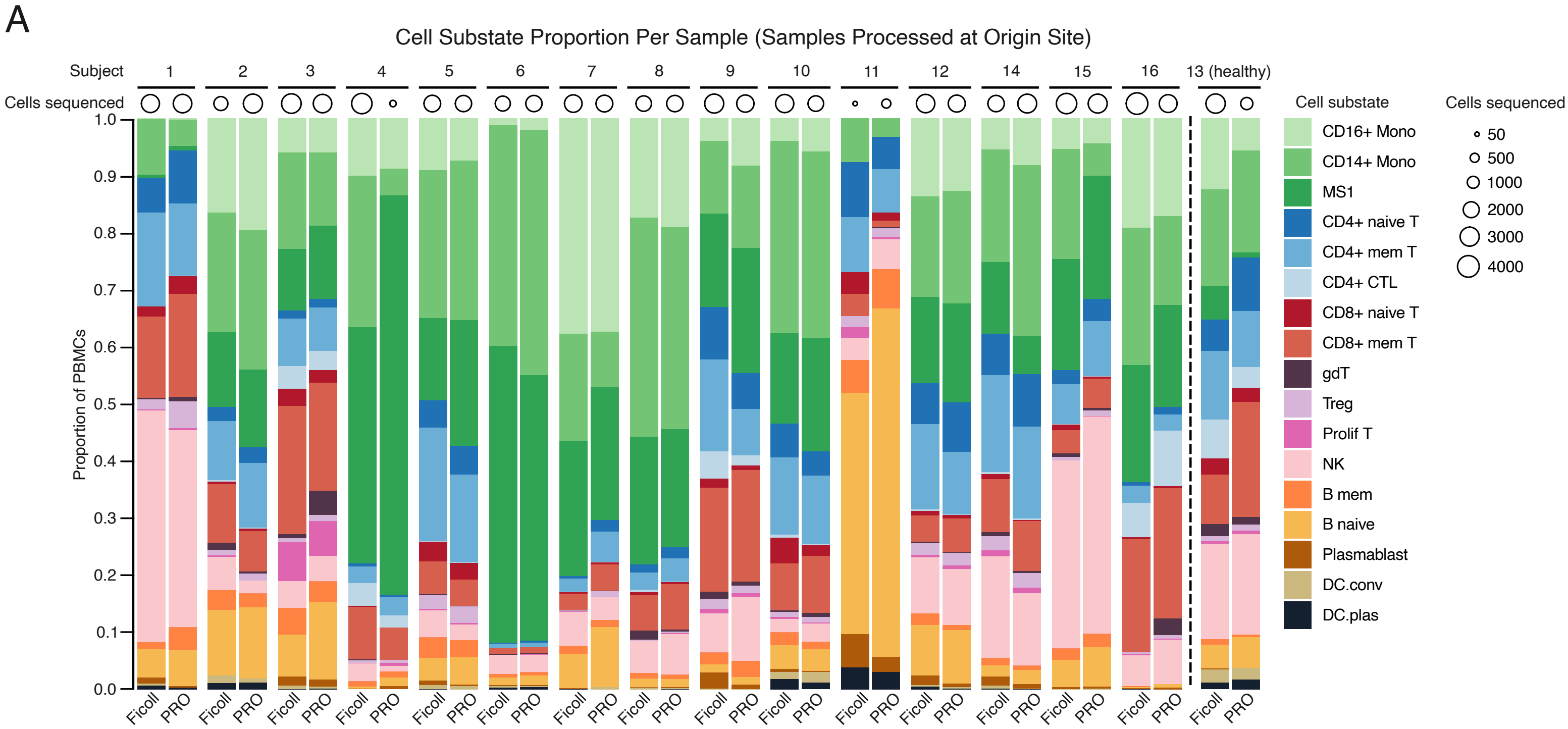

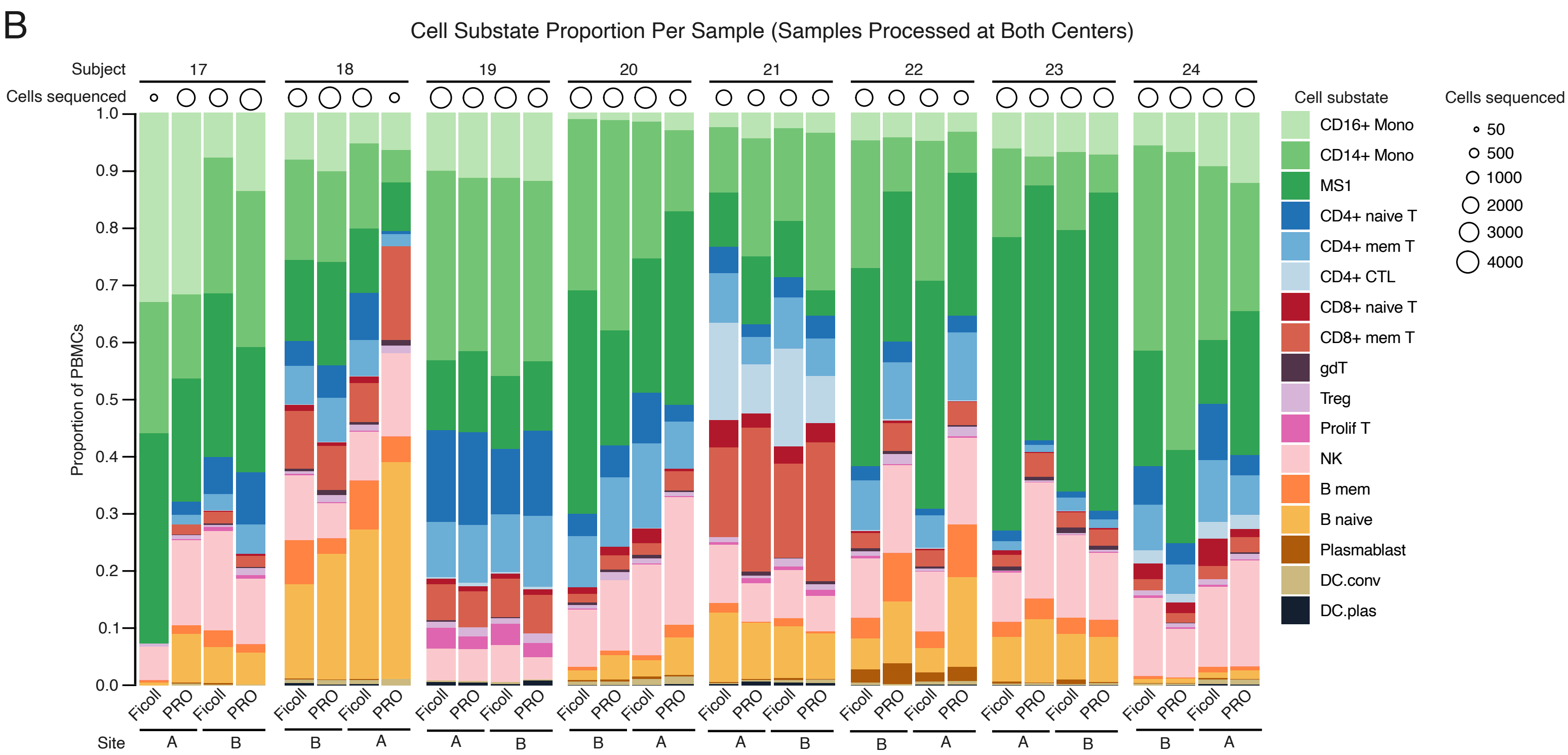

Supplemental Figure 5. Cell substate proportions for samples processed at single centers **(A)** and technical duplicate samples processed at both centers **(B)**. Samples from the same patient processed using different methods are shown next to each other. In (B), the corresponding pair of technical duplicates processed at the non-origin site are shown subsequently, with the labeled site indicating where each sample was processed. PRO denotes Cryo-PRO.

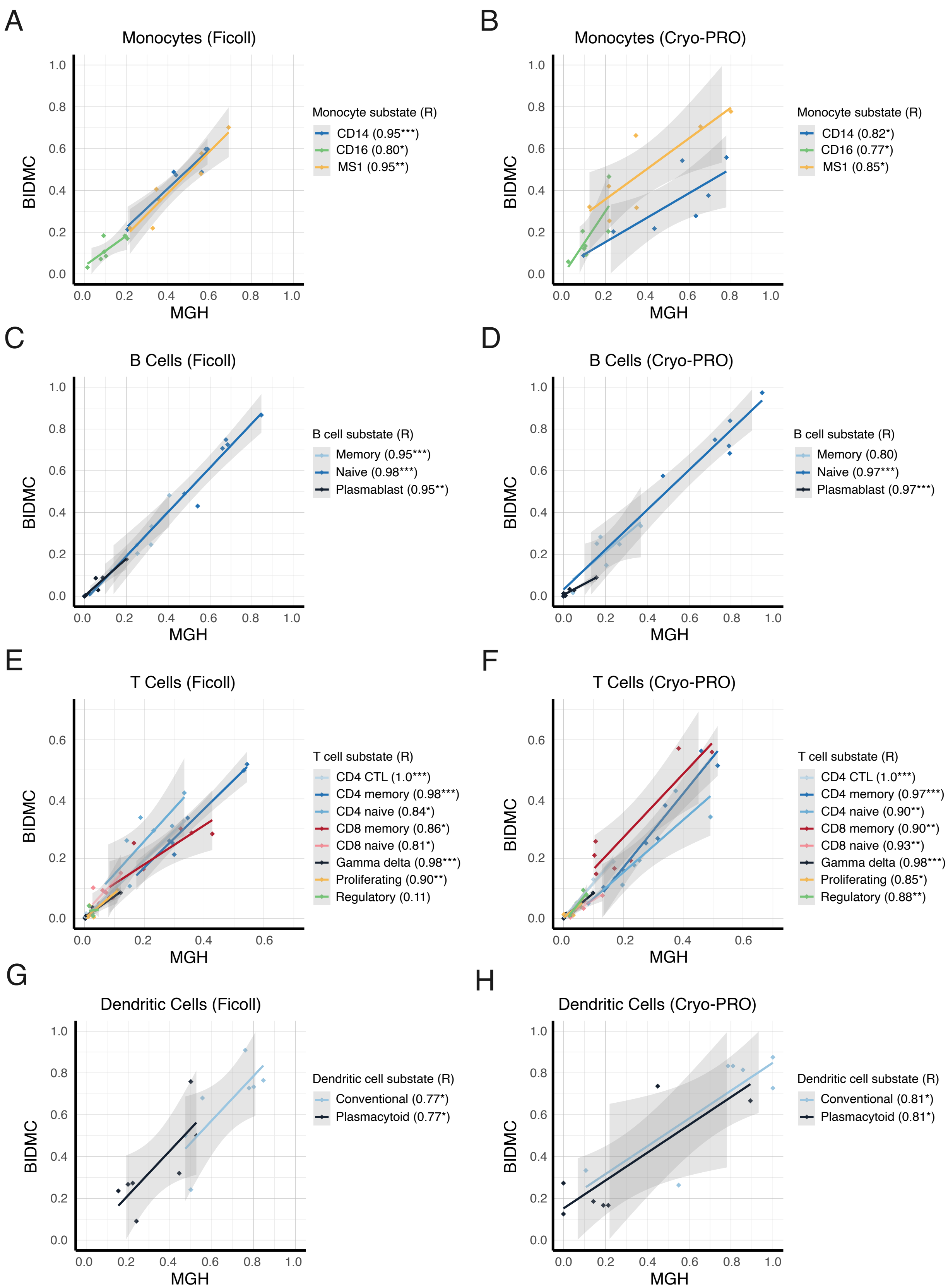

Supplemental Figure 6. Scatter plots of cell substate proportions from different processing sites. Each cell substate is represented by a different color. Proportion is the number of cellsof one cell substate divided by the total number of cells from its cell type from that patient sample. The patient-paired Ficoll:Ficoll samples and Cryo-PRO:Cryo-PRO samples from the two different enrollment sites are plotted to assess correlation of technical duplicates for each patient. Trendlines indicate a linear regression fit with shaded 95% confidence intervals. Pearson's correlations (R, *p < 0.05, ** p < 0.01, ***p < 0.001) are shown for all correlations.

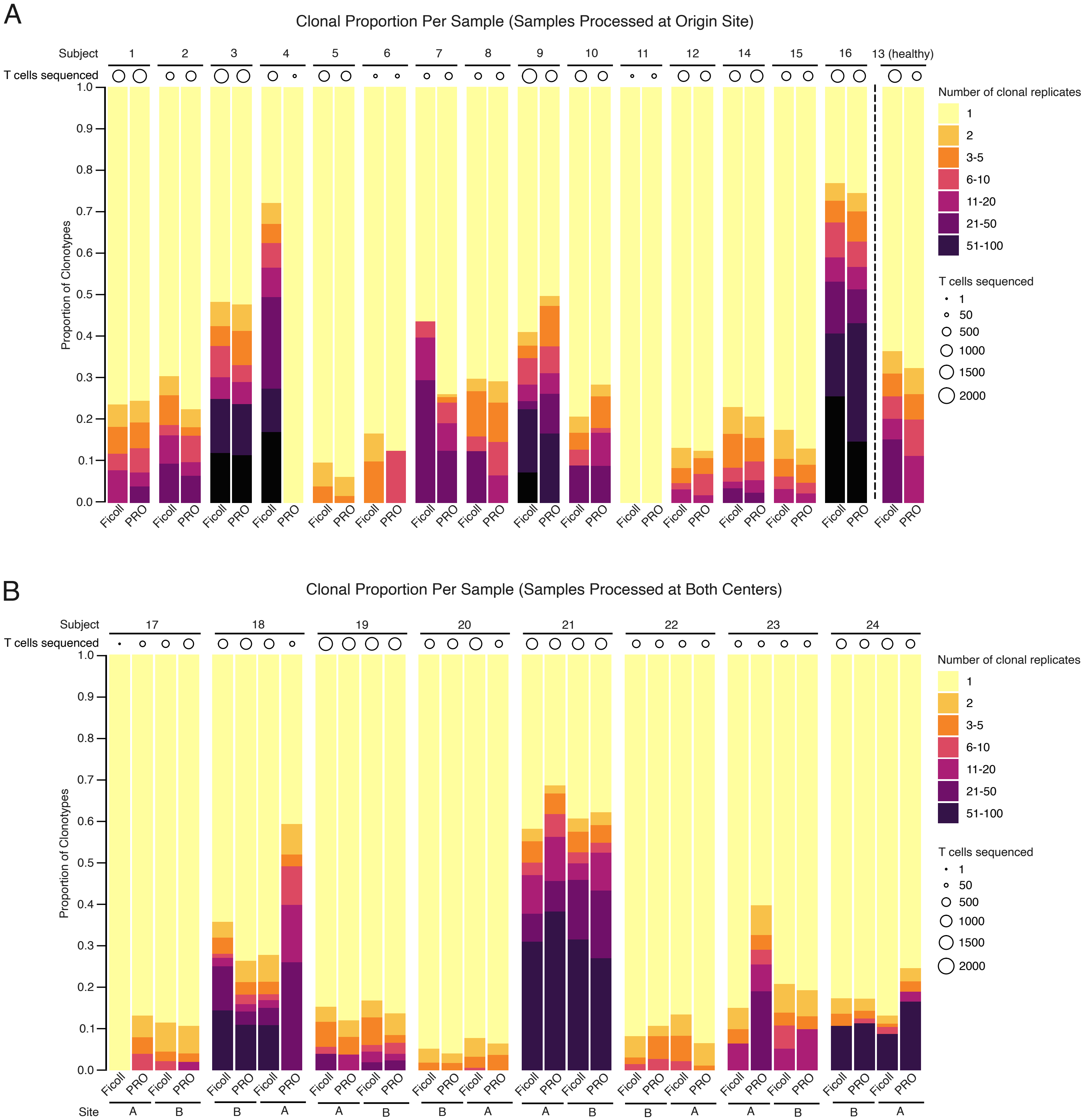

Supplemental Figure 7. Clonal expansion proportions for samples processed at single centers **(A)** and technical duplicate samples processed at both centers **(B)**. Samples from the same patient processed using different methods are shown next to each other. In (B), the corresponding pair of technical duplicates processed at the non-origin site are shown subsequently, with the labeled site indicating where each sample was processed. PRO denotes Cryo-PRO.

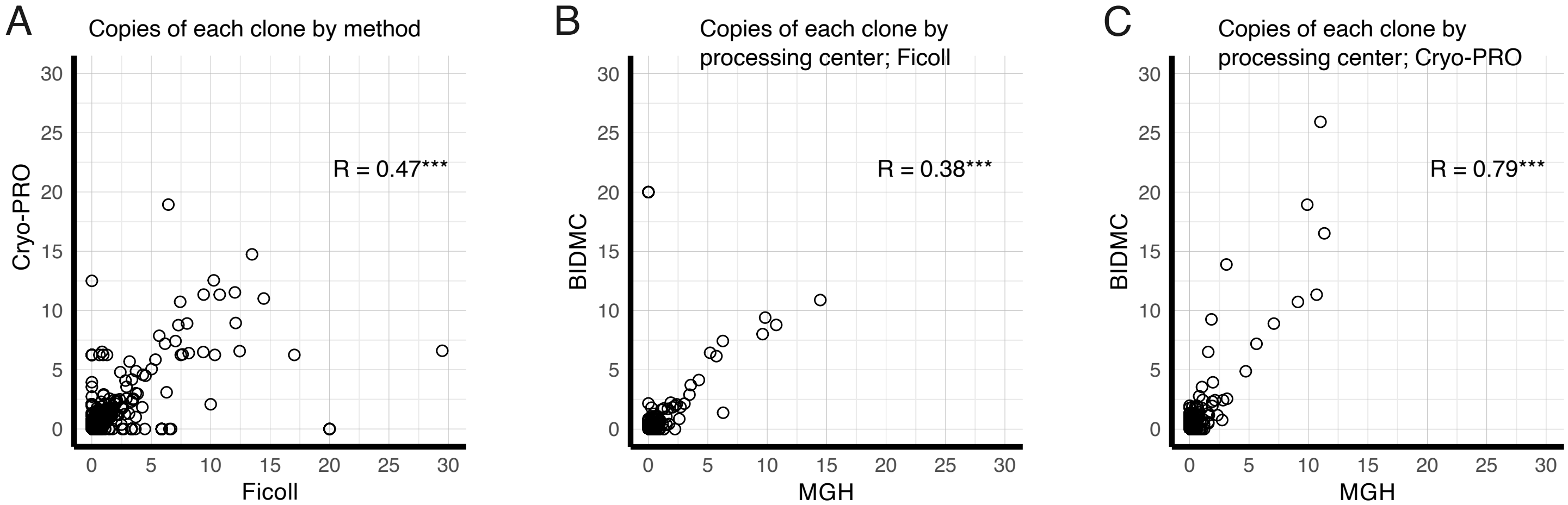

Supplemental Figure 8. Scatter plot of the clonotype sequence frequency for all samples processed by different methods at single centers **(A)** and for technical duplicate samples processed at both centers by Ficoll **(B)** and Cryo-PRO **(C)**. The proportion of each unique sequence out of total TCR transcripts in each sampleis plotted against its corresponding sample to compare similarities in Ficoll vs Cryo-PRO (A), or in processing center (B,C). Pearson's correlations (R, $*p < 0.05$, $** p < 0.01$, $***p < 0.001$) are shown for all correlations.

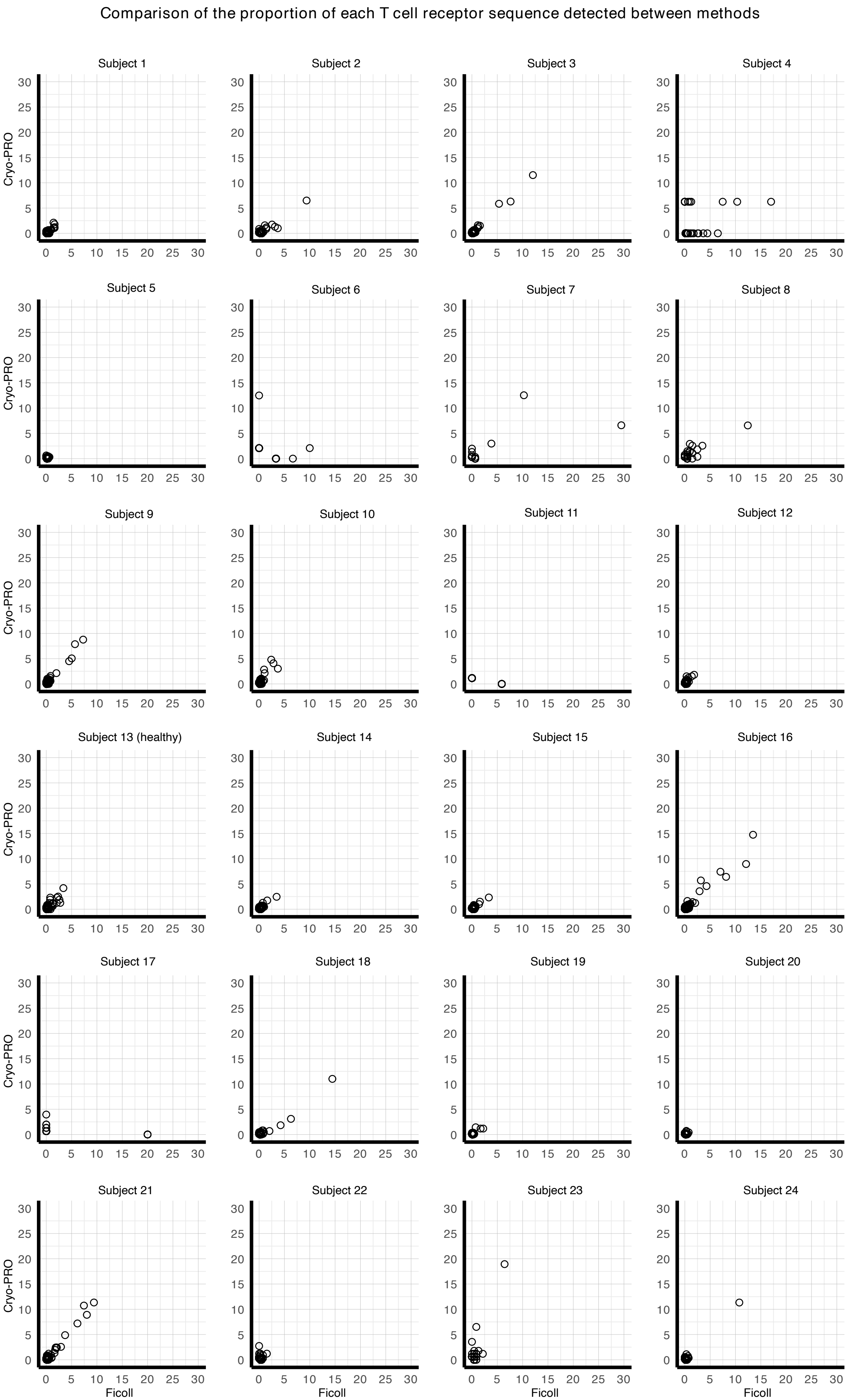

Supplemental Figure 9. Scatter plot of clonotype sequence frequency for all samples processed by different methods at single centers, individually plotted for each subject. The proportion of each unique sequence out of total TCR transcripts in each Ficoll sample is plotted against its corresponding Cryo-PRO sample to compare similarities in TCR clonal proportion.

Comparison of the proportion of each T cell receptor sequence detected between processing centers

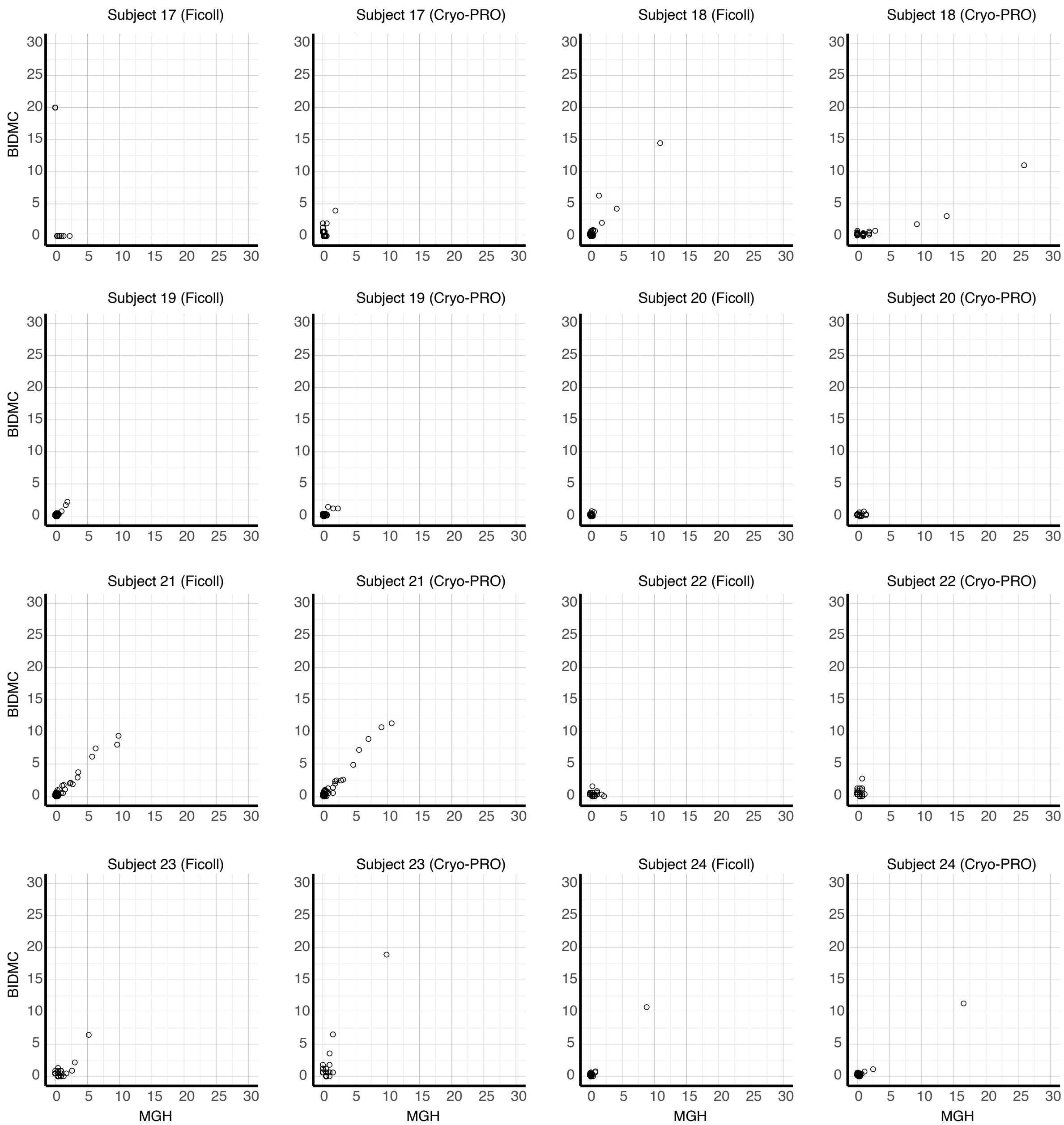

Supplemental Figure 10. Scatter plot of the clonotype sequence frequency for technical duplicate samples processed at both centers by Ficoll (left) and Cryo-PRO (right), individually plotted for each subject. The proportion of each unique sequence out of total TCR transcripts in each MGH sample is plotted against its corresponding BIDMC sample to compare similarities in TCR clonal proportion.
